# Programmable Nanobody‐Targeting Chimeras Enable Intracellular Viral Protein Degradation

**DOI:** 10.1002/advs.76687

**Published:** 2026-07-17

**Authors:** Max Yu‐Chen Pan, Ting‐Hui Lee, Tzu‐Ning Peng, Eva Yi‐Hsuan Wu, Yu‐Ting Chiu, Chih‐Chien Chiu, Hong Vinh Nguyen, Yuan‐Shao Pao, Ming‐Hong Chao, Richard Kuan‐Lin Lee, Han‐Chung Wu, Lily Hui‐Ching Wang

**Affiliations:** ^1^ Institute of Molecular and Cellular Biology National Tsing Hua University Hsinchu Taiwan; ^2^ SMOBIO Technology, Inc. Hsinchu Taiwan; ^3^ Institute of Cellular and Organismic Biology Academia Sinica Taipei Taiwan; ^4^ School of Medicine National Tsing Hua University Hsinchu Taiwan; ^5^ MS in Regulatory Affairs for Drug & Medical Devices National Tsing Hua University Hsinchu Taiwan

**Keywords:** bio‐PROTAC, hepatitis B virus, Nab‐TAC, proximity‐induced degradation, targeted protein degradation

## Abstract

Chronic hepatitis B virus (HBV) infection remains a major global health challenge, largely because current antiviral therapies seldom achieve sustained clearance of the viral surface antigen (HBsAg), a central driver of immune tolerance and viral persistence. Here, we present a programmable, nanobody‐based targeted protein degradation platform, termed Nanobody‐Targeting Chimera (Nab‐TAC), designed for the intracellular clearance of HBV antigens. To systematically identify functional degradation modules, we developed a split‐luciferase complementation‐based quantitative screening platform that benchmarks degron motifs and proximity‐inducing effectors, enabling comprehensive ranking of proteasome‐ and lysosome‐targeting elements across diverse cellular contexts and subcellular compartments. By coupling phage display‐derived nanobodies and single‐chain variable fragments with these optimized degradation modules, we engineered a panel of Nab‐TAC constructs that efficiently degrade viral HBsAg in hepatocytes. Notably, the lead construct, E3‐FCGR3B, achieved robust intrahepatic antigen clearance and markedly reduced circulating HBsAg levels in an HBV hydrodynamic injection mouse model. Together, these findings establish Nab‐TAC as a modular and versatile platform for targeted antiviral protein degradation and highlight its potential as a therapeutic strategy toward a functional cure for chronic HBV infection.

## Graphical abstract

Nab‐TAC enables targeted degradation of HBV surface antigen (HBsAg) in vivo. By fusing nanobody‐based recognition domains with programmable proteasome‐recruiting degradation signals, Nab‐TAC reduces HBsAg levels in a hydrodynamic HBV mouse model.

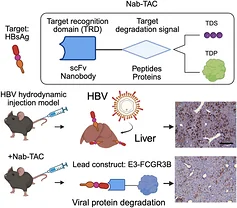

## 1 Introduction

Chronic hepatitis B virus (HBV) infection affects an estimated 254 million people worldwide and remains a major cause of liver cirrhosis and hepatocellular carcinoma [1]. The HBV life cycle involves hepatocyte entry via the sodium taurocholate cotransporting polypeptide receptor, nuclear conversion of relaxed circular DNA into covalently closed circular DNA (cccDNA), and production of viral RNAs and proteins from this stable nuclear template. Hepatitis B surface antigen (HBsAg) comprises three structurally related forms: the large (LHBS), middle (MHBS), and small (SHBS) hepatitis B surface antigens, which share a common S domain but differ in their N‐terminal preS1 and preS2 extensions. HBsAg is produced in large excess during viral replication and secreted as both virion‐associated and non‐infectious subviral particles [2]. Persistent HBsAg expression arises not only from cccDNA but also from integrated HBV DNA, allowing ongoing antigen production independent of active viral replication [3, 4, 5, 6].

HBsAg loss is considered a key marker of functional cure but is rarely achieved with current therapies [7, 8]. Current first‐line therapies for chronic HBV, including nucleos(t)ide analogs and pegylated interferon‐α, effectively suppress serum HBV DNA in most patients; however, the degree of HBsAg loss remains limited [9]. Systematic analyses report annual functional cure rates well below 1% with NA therapy alone, and although interferon‐α can modestly improve HBsAg clearance, sustained HBsAg seroclearance is still rare even after prolonged treatment [10]. Persistent high HBsAg levels drive HBV‐specific immune dysfunction by impairing T‐ and B‐cell responses, promoting immune tolerance, and increasing the risk of relapse after treatment discontinuation [11]. As a structural protein lacking enzymatic activity, HBsAg is poorly amenable to conventional small‐molecule inhibition, placing it among the class of “undruggable” targets [12].

Targeted protein degradation (TPD) has emerged as a therapeutic paradigm that leverages cellular quality control systems, particularly the ubiquitin‐proteasome system (UPS), to eliminate disease‐causing proteins rather than merely inhibiting them, extending the druggable proteome to targets lacking classical binding pockets [13, 14, 15]. One of the most established TPD modalities is the proteolysis‐targeting chimera (PROTAC), a bifunctional small molecule that recruits an E3 ubiquitin ligase to a target protein, triggering its ubiquitination and proteasomal degradation. This event‐driven mechanism enables sustained depletion of the target protein and can modulate proteins that are traditionally considered difficult to target [16, 17]. However, classical PROTACs require high‐affinity ligands for both the target and the recruited E3 ligase, a condition rarely met for secretory proteins such as HBsAg [18, 19]. On the other hand, biological PROTACs (bioPROTACs) address this limitation by replacing small‐molecule recognition elements with biologic modules, such as peptides, antibodies, or nanobodies, enabling higher affinity and specificity toward challenging targets [19, 20].

In this study, we develop a nanobody‐based targeted protein degradation platform, termed Nanobody‐Targeting Chimera (Nab‐TAC), that combines a single‐domain antibody with a target degradation signal (TDS) to engage intracellular degradation pathways and directly eliminate HBsAg. By integrating nanobody‐mediated target recognition with systematically optimized degradation modules, Nab‐TAC addresses the unmet therapeutic challenge of persistent antigen burden in chronic HBV infection.

## 2 Results

### 2.1 Selection Of Target Degradation Signals

To establish a robust and quantitative platform for identifying functional TDS, we employed the NanoLuc Binary Technology (NanoBiT) system, in which a Large BiT (LgBiT) subunit complements a high‐affinity HiBiT peptide to generate luminescence [21]. The HiBiT tag was fused to 14 candidate TDS peptides curated from the literature, comprising 8 proteasome‐targeting (TDS #1∼8; cIAP, Keap1, VHL, IκBα, and TRIM7 ligands) and 6 lysosome‐targeting elements (TDS #9∼14; Nix/LC3, LIMP‐II, CI‐MPR, p62/LC3, CMA, and NPGY motifs) (Table 1) [22, 23, 24, 25, 26, 27, 28, 29, 30, 31, 32, 33, 34, 35, 36, 37, 38, 39]. In this design, TDS‐mediated degradation of the HiBiT fusion reduces LgBiT‐HiBiT complementation, providing a luminescence‐based readout of intracellular protein stability. The platform was deployed in both HeLa and HepG2 cells (Figure 1a). To calibrate assay sensitivity, HiBiT‐SMAC containing a SMAC‐derived ligand, a well‐characterized proteasome‐recruiting degrader [22, 23], was used as a positive control. Increasing the HiBiT‐SMAC:LgBiT molar ratio (3‐, 4‐, 5‐, and 10‐fold) produced a dose‐dependent decrease in luminescence in HeLa (48 h) and HepG2 (72 h) cells upon transient transfections (Figure 1b,c), with a 5‐fold excess yielding the most reproducible dynamic range and the strongest response in HepG2 at 72 h (Figure 1d,e); this condition was therefore adopted for subsequent screens.

**TABLE 1:** Target degradation signals (TDS) employed in this study and their intracellular degradation mechanisms.

TDS	Peptide name	Specificity
1	SMAC ligand	Collin E3 ligase cIAP recognition motif [22, 23]
2	Keap1	Collin RING E3 ligase Keap‐1 recognition motif [24]
3	VHL	Collin‐1 E3 ligase VHL recognition motif (HIF1a) [25, 26, 27, 28]
4	IκBα phosphopeptide	Collin E3 ligase recognition motif [28, 29]
5	CVB3 2C ligand	Exogenous TRIM7 recognition motif [30, 31]
6	Norovirus NSP 5 ligand	Exogenous TRIM7 recognition motif [30]
7	Norovirus NSP 8 ligand	Exogenous TRIM7 recognition motif [30]
8	RWDD2B ligand	Endogenous TRIM7 recognition motif [30]
9	Nix LC3	Nix/ LC3 interaction motif [32]
10	Lysosome sorting signal (LIMP‐II)	LIMP‐II lysosome sorting signal [33, 34]
11	CI‐MPR Acidic‐cluster‐dileucine sorting‐signal	CI‐MPR lysosome sorting signal [33, 34]
12	p62 LC3	p62/ LC3 interaction motif [34, 35]
13	CMA motif	Chaperon 70 mediated lysosomal derogation signal [36, 37]
14	NPGY	Lysosome sorting motif [38, 39]

**FIGURE 1:**
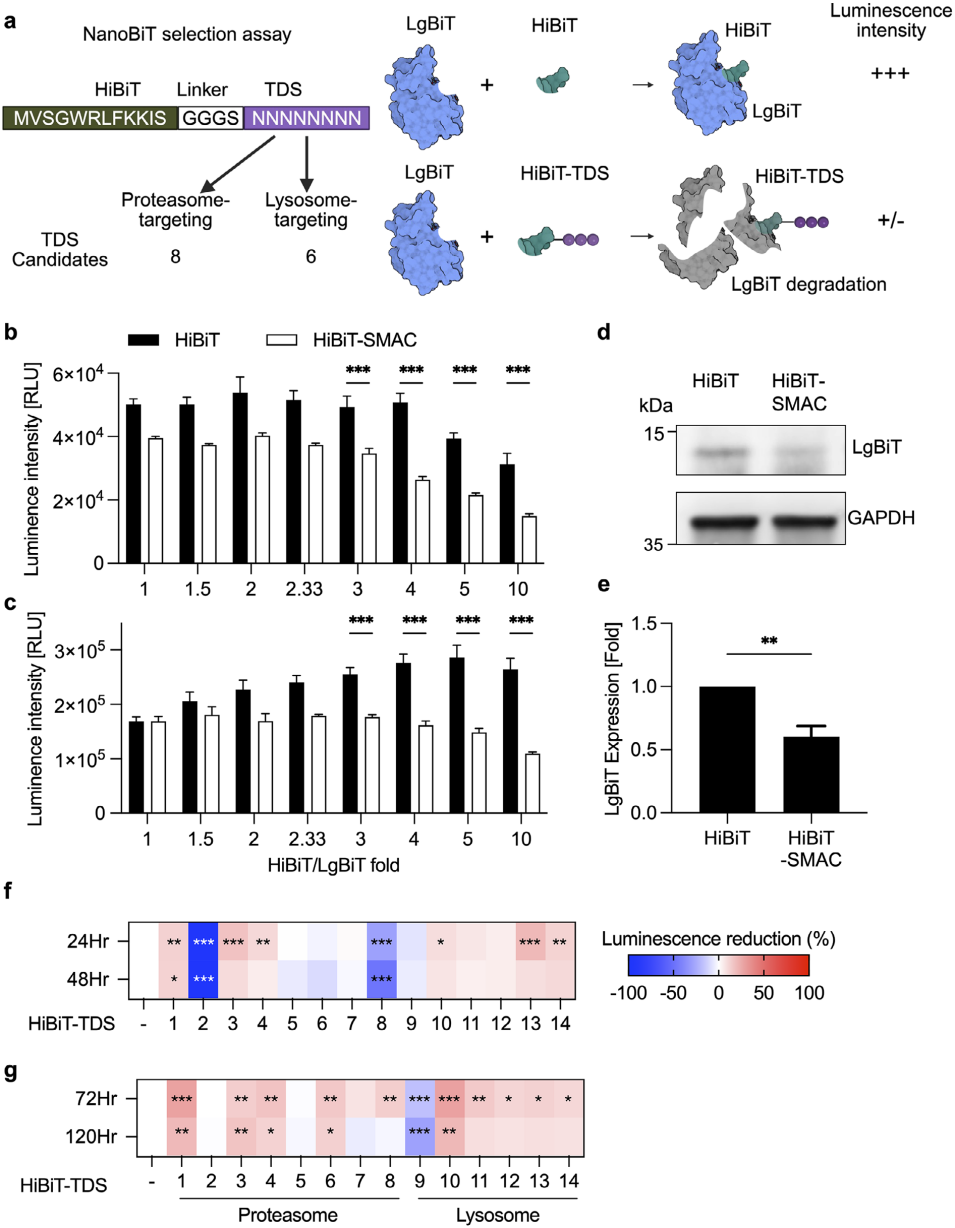
Establishment and validation of a NanoBiT‐based intracellular protein stability selection platform. (a) Schematic illustration of the NanoBiT‐based assay for quantitative evaluation of intracellular protein stability via LgBiT/HiBiT complementation. (b,c) Luminescence signals measured in HeLa (48 h) and HepG2 (72 h) cells following co‐transfection of LgBiT with HiBiT‐SMAC constructs at increasing HiBiT/LgBiT ratios. (d) Representative blots of HiBiT‐SMAC and control constructs in HepG2 cells, confirming protein expression levels. (e) Densitometric quantification of Western blot signals shown in (d). Band intensities were normalized to the corresponding loading control and expressed as fold change relative to mock. Data are presented as mean ± SEM. (f,g) Heat map representation of luminescence change in HeLa (24 h, 48 h) and HepG2 (72 h, 120 h) cells following expression of target degradation signal (TDS) candidates. Data are presented as percentage change (−100% to 100%) relative to control, illustrating differential degradation efficiency across candidates and time points. Data are representative of at least two independent experiments. All experiments were performed in biological triplicates (n = 3). Data in (e) were analyzed using an unpaired two‐tailed Student's t‐test. Data in (b) and (c) were analyzed by two‐way ANOVA followed by Šídák's multiple comparisons test. Data in (f) and (g) were analyzed by ordinary one‐way ANOVA followed by Dunnett's multiple comparisons test. Statistical significance is indicated as follows: ∗*p* < 0.05; ∗∗*p* < 0.01; ∗∗∗*p* < 0.001; ns, not significant. Statistical analyses were conducted using GraphPad Prism 10.

Screening of the 14 TDS candidates revealed distinct cell‐line‐dependent activity profiles. In HeLa cells, TDS #1 induced consistent luminescence reduction at both 24 and 48 h, while TDS #7 and #14 increased luminescence, suggesting impaired degradation activity (Figure 1f; corresponding numerical values listed in Tables S1 and S2). In HepG2 cells, TDS #1, #3, #4, #6, and #10 showed significant luminescence reduction at 72 and 120 h post‐transfection (Figure 1g, Tables S3 and S4). We suggest that the differential activity between cell lines reflects the well‐established dependence of degrader efficacy on intracellular E3 ligase and autophagy receptor abundance and is therefore interpreted as a mechanistically informative feature of the screening data rather than a platform limitation [23, 40].

### 2.2 Establish Proximity‐based Protein Degradation Using Nanobody‐guided Degradation Assay

Building on a prior proteome‐scale study that identified degradation and stabilization effectors using an anti‐EGFP nanobody (vhhGFP) platform [41], we sought to expand the repertoire of degradation‐inducing modules by evaluating proximity‐based targeted degradation proteins (TDPs, Table 2) [39, 41, 42]. Each TDP was fused to an N‐terminal HiBiT tag and co‐transfected with LgBiT in HeLa and HepG2 cells, allowing target degradation to be quantified as luminescence signal reduction. In HeLa cells, all TDP candidates induced significant luminescence reduction, indicating robust degradation activity. In HepG2 cells, the same trend was observed except for UBE2B and GABARAP, which failed to produce a significant signal decrease (Figure 2a; Tables S5 and S6). Western blot analysis further confirmed marked reduction of LgBiT in HepG2 expressing HiBiT‐FCGR3B, SPOP, LY6D, GABARAP, and EID1 (Figure 2b,c; Table S7). A comparable reduction of LgBiT protein was also observed in HeLa cells expressing the same HiBiT‐TDP constructs (Figure S1a,b; Table S8).

**TABLE 2:** Target degradation proteins (TDP) employed in this study and their intracellular degradation mechanisms.

TDP	Pathway	Protein name	Specificity
1	UPS	UBE2B	E2 ubiquitin conjugating enzymes [41]
2	UPS	FBXL12	Adaptor of CRL1/SCF [41]
3	UPS	FBXL15	Adaptor of CRL1/SCF [41]
4	UPS	SPOP	Substrate recognition protein of CRL3 [41, 42]
5	UPS	EID1	C‐terminal degron sequence [41]
6	UPS	PRR20A	C‐terminal degron sequence [41]
7	unclassified	FCGR3B	GPI‐Anchored protein [41]
8	unclassified	LY6D	GPI‐Anchored protein [41]
9	Lysosome	GABARAP	Lysosome related protein [41]

**FIGURE 2:**
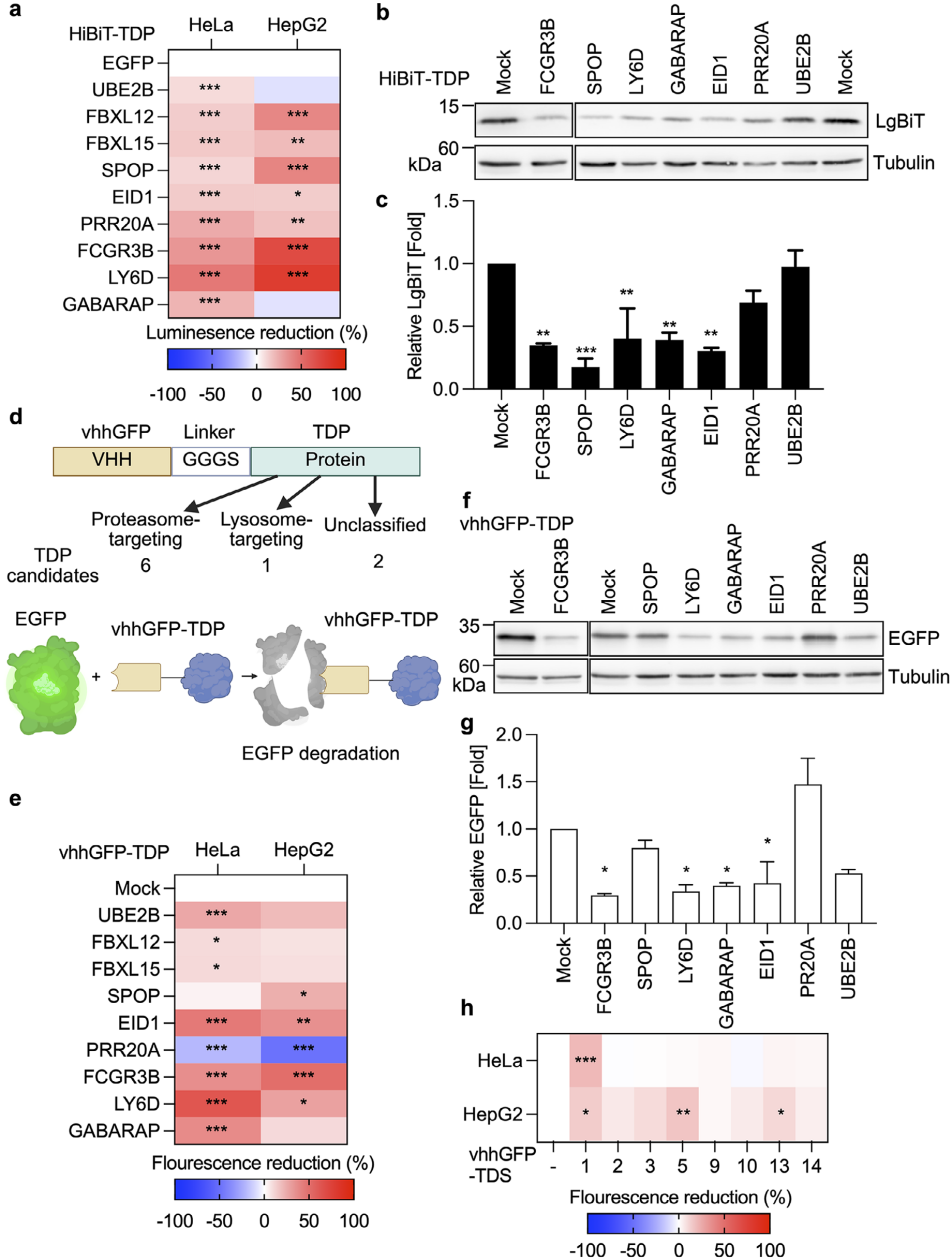
Functional screening and validation of proximity‐based target degradation proteins (TDPs). (a) Heat map summarizing luminescence change mediated by HiBiT‐TDP constructs in HeLa and HepG2 cells at 72 h. (b) Representative blots of TDP candidates (HiBiT‐FCGR3B, LY6D, GABARAP, EID1, and SPOP) compared with HiBiT control in HepG2 cells. (c) Densitometric quantification of Western blot signals shown in (b). Band intensities were normalized to the corresponding loading control and expressed as fold change relative to mock. Data are presented as mean ± SEM. (d) Schematic illustration of the anti‐GFP nanobody (vhhGFP)‐based degradation assay for fluorescence‐based validation of TDP activity. (e) Heat map showing percentage change in enhanced green fluorescent protein (EGFP) fluorescence intensity mediated by vhhGFP‐TDP constructs in HeLa and HepG2 cells at 72 h. (f) Representative blots of EGFP levels in HepG2 cells expressing vhhGFP‐TDP constructs. (g) Densitometric quantification of Western blot signals shown in (f). Band intensities were normalized to the corresponding loading control and expressed as fold change relative to mock. Data are presented as mean ± SEM. (h) Comparative fluorescence reduction induced by vhhGFP‐TDS constructs at 48 h in both cell lines. Heat maps in (a), (e), and (h) display percentage change (−100% to 100%) relative to respective controls. Data are representative of at least two independent experiments. All experiments were performed with three independent biological replicates (n = 3). Data in (c) were analyzed using an unpaired two‐tailed Student's t‐test. Data in (a), (e), (g), and (h) were analyzed by ordinary one‐way ANOVA followed by Dunnett's multiple comparisons test. Statistical significance is indicated as follows: ∗*p* < 0.05; ∗∗*p* < 0.01; ∗∗∗*p* < 0.001; ns, not significant. Statistical analyses were conducted using GraphPad Prism 10.

To validate that these TDPs can drive degradation of an independent target, we engineered modular fusions consisting of N‐terminal vhhGFP and C‐terminal TDPs (Figure 2d) and assessed EGFP fluorescence as a readout of protein abundance. In both HeLa and HepG2 cells, vhhGFP fusions with EID1, FCGR3B, and LY6D produced significant EGFP reductions (Figure 2e; Tables S9 and S10). Western blot analysis further confirmed substantial EGFP depletion in HepG2 cells expressing vhhGFP‐GABARAP, ‐FCGR3B, ‐LY6D, and ‐EID1, except for PRR20A (Figure 2f,g; Table S11). A comparable reduction of EGFP protein was observed in HeLa cells expressing the same vhhGFP‐TDP constructs (Figure S1c,d; Table S12). In parallel, we tested whether vhhGFP could be directly coupled to previously identified TDS motifs. In HepG2 cells, vhhGFP‐TDS #1, #5, and #13 produced significant EGFP reductions, whereas in HeLa cells only TDS #1 reached statistical significance (Figure 2h, Tables S13 and S14). Together, these data underscore the influence of cellular context on degradation efficacy and demonstrate that both proximity‐based TDPs and peptide‐based TDS elements can be leveraged for nanobody‐guided targeted protein degradation.

Across all TDP candidates evaluated, FCGR3B consistently produced the most pronounced degradation in both HeLa and HepG2 cells, identifying it as the most robust effector among the screened modules. To dissect the mechanism underlying this strong activity, we performed inhibitor‐based pathway validation. Treatment with the proteasome inhibitor MG132 markedly restored the NanoBiT luminescence signal in cells expressing HiBiT‐FCGR3B, whereas lysosome inhibitors (bafilomycin A1, chloroquine, and U18666A) had minimal effects (Figure S2a). Consistently, in the vhhGFP‐based assay, MG132 substantially rescued EGFP fluorescence in cells expressing vhhGFP‐FCGR3B, while lysosome inhibition produced no comparable rescue (Figure S2b). Western blot analysis further confirmed proteasome‐dependent rescue of EGFP protein levels in vhhGFP‐FCGR3B‐expressing cells (Figure S2c,d). Collectively, these inhibitor‐based mechanistic controls establish that FCGR3B‐mediated degradation is predominantly proteasome‐dependent rather than lysosome‐mediated. Together, the proteome‐scale screen and orthogonal validation establish nanobody‐directed proximity degraders as a versatile strategy and identify multiple TDP candidates suitable for downstream Nab‐TAC construction.

### 2.3 Evaluation Of Nuclear Protein Degradation Using LgBiT‐EGFP‐NLS Reporter System

To evaluate whether targeted degradation strategies can be applied to nuclear proteins, we engineered a dual‐reporter LgBiT‐EGFP‐NLS fusion that enables simultaneous luminescence and fluorescence readouts of protein abundance. The nuclear localization signal (NLS) confined the reporter predominantly to the nucleus (Figure 3a), and a stable HeLa cell line (HeLa‐LgBiT‐EGFP‐NLS) was generated for downstream assays. We first assessed HiBiT‐based proximity‐induced degraders. Luminescence measurements showed significant reductions across all candidates, indicating efficient engagement with the LgBiT‐tagged reporter. However, fluorescence‐based protein abundance was significantly reduced only by FBXL12, FBXL15, SPOP, FCGR3B, and LY6D (Figure 3b, Tables S15 and S16). We suggest that this discordance between the two readouts is attributable to steric interference from specific TDP modules on the HiBiT tag, which lowers HiBiT‐LgBiT complementation independently of actual protein degradation. Consistently, UBE2B, GABARAP, EID1, and PRR20A showed significant luminescence loss without a corresponding fluorescence decrease (Tables S15 and S16), reflecting TRD‐size‐dependent complementation interference rather than genuine protein depletion. Consistent with fluorescence quantification, immunofluorescence imaging further confirmed substantial reductions in nuclear EGFP signal in cells expressing HiBiT‐SPOP and HiBiT‐FCGR3B (Figure 3c,d), supporting their ability to mediate nuclear protein degradation.

**FIGURE 3:**
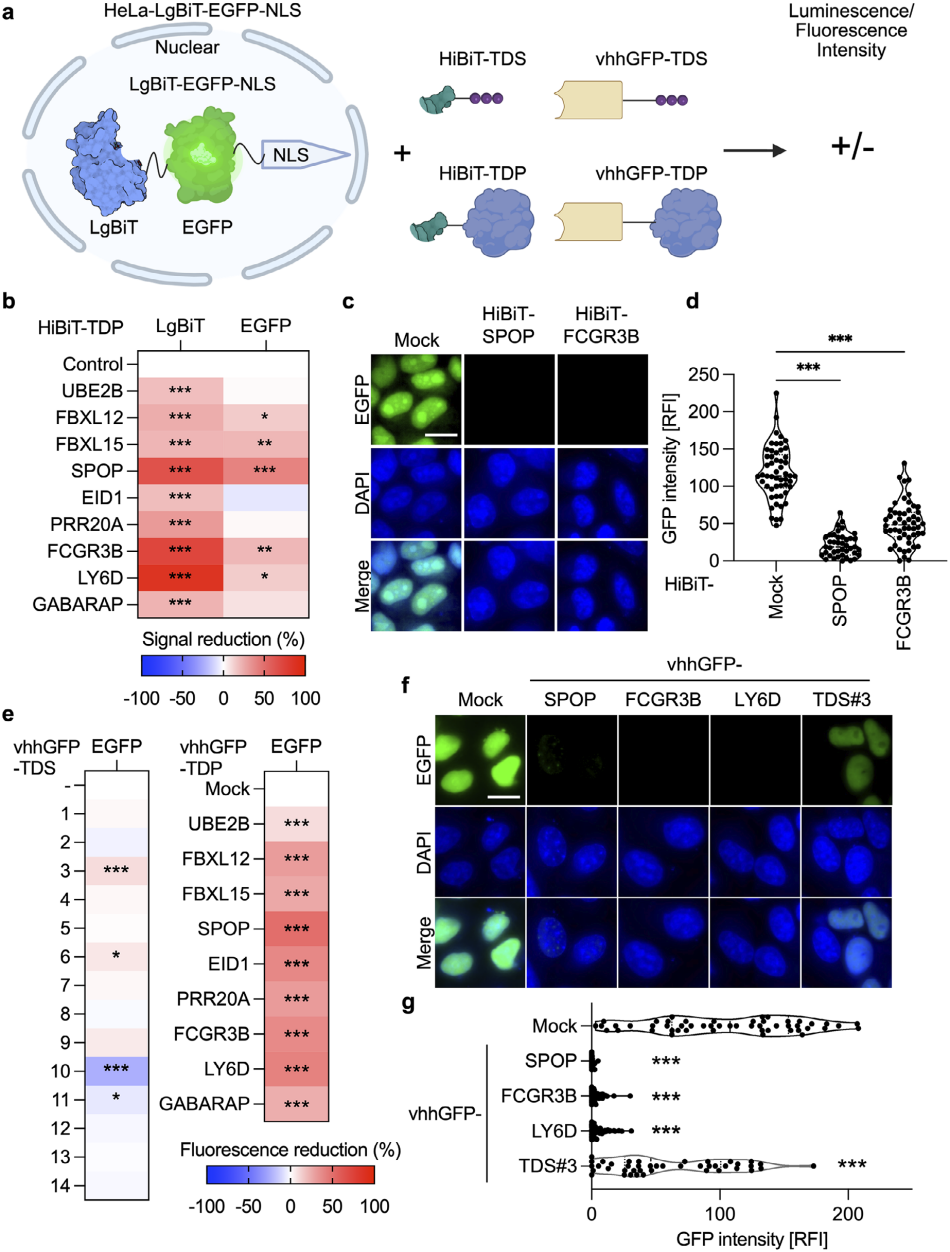
Functional screening and orthogonal validation of candidate TDP and TDS modules for nuclear protein degradation. (a) Schematic representation of the dual‐reporter system combining NanoBiT‐based luminescence and fluorescence readouts in a HeLa‐LgBiT‐EGFP‐nuclear localization signal (NLS) stable cell line, enabling quantitative assessment of nuclear protein degradation. (b) Heat map summarizing percentage change in luminescence and fluorescence signals induced by HiBiT‐TDP constructs in the HeLa‐LgBiT‐EGFP‐NLS cell line at 48 h post‐transfection. (c) Representative fluorescence microscopy images of nuclear EGFP signal in HeLa‐LgBiT‐EGFP‐NLS cells expressing HiBiT‐SPOP and HiBiT‐FCGR3B, demonstrating reduced nuclear fluorescence intensity compared with control. Scale bar, 20 µm. (d) Quantification of nuclear EGFP fluorescence intensity corresponding to (c). A total of 100 cells per condition were analyzed across three independent experiments. Data are presented as mean ± SEM. (e) Heat map depicting percentage change in fluorescence mediated by vhhGFP‐TDP and vhhGFP‐TDS constructs in the HeLa‐LgBiT‐EGFP‐NLS cell line at 48 h. (f) Representative fluorescence microscopy images showing nuclear EGFP attenuation following expression of vhhGFP‐SPOP, vhhGFP‐FCGR3B, and vhhGFP‐LY6D constructs in HeLa‐LgBiT‐EGFP‐NLS. Scale bar, 20 µm. (g) Quantitative analysis of nuclear EGFP fluorescence corresponding to (f), based on 100 cells per condition from three independent experiments. Data are presented as mean ± SEM. Heat maps in (b) and (e) display percentage change (−100% to 100%) relative to the control. Data are representative of at least two independent experiments. All experiments were performed with three independent biological replicates (n = 3). Data in (b) and (e) were analyzed using ordinary one‐way ANOVA followed by Dunnett's multiple comparisons test. Data in (d) and (g) were analyzed using Brown‐Forsythe and Welch's ANOVA followed by Dunnett's T3 multiple comparisons test. Statistical significance is indicated as follows: ∗*p* < 0.05; ∗∗*p* < 0.01; ∗∗∗*p* < 0.001; ns, not significant. Statistical analyses were performed using GraphPad Prism 10.

We next evaluated vhhGFP‐based constructs targeting the same nuclear reporter. When the four discordant candidates (UBE2B, GABARAP, EID1, and PRR20A) were re‐tested as vhhGFP‐TDP fusions, all four produced significant fluorescence reduction (Figure 3e, Table S18), confirming that their degradation capacity is intact and that the prior discordance reflects steric interference of bulky TDP modules with the HiBiT system rather than an absence of degradation activity. Because the vhhGFP readout scores degradation through EGFP loss independently of HiBiT‐LgBiT complementation, it bypasses the steric constraint of the dual reporter and recovers the true degradation signal for these candidates. Among vhhGFP‐TDS candidates, only TDS #3 and TDS #6 achieved statistically significant reductions (Figure 3e, Tables S17), indicating comparatively limited activity of peptide‐based TDS elements in the nuclear context. Immunofluorescence imaging further supported these findings, with marked nuclear EGFP depletion in cells expressing vhhGFP‐SPOP, ‐FCGR3B, and ‐LY6D (Figure 3f,g).

### 2.4 Development and Validation of anti‐SHBS and Anti‐Lhbs Nab‐TAC

To identify suitable target recognition domains (TRDs) for HBV surface antigens, we performed phage display screening of alpaca‐derived nanobody and human antibody libraries, identifying six anti‐HBsAg VHH sequences and two anti‐PreS1 scFv clones with high binding potential (Figure 4a). Here, TDS #1, #3, #10, #13, and #14 were selected based on consistent and significant degradation activity in at least two assay systems across both cell lines (Figure 1f,g, 2h, 3e; Tables S1–S4,S13‐S14,S17), and were combined with the top‐performing TRDs, including scFv H1 and VHH E3 and H3, to generate a panel of TDS‐based Nab‐TAC constructs.

**FIGURE 4:**
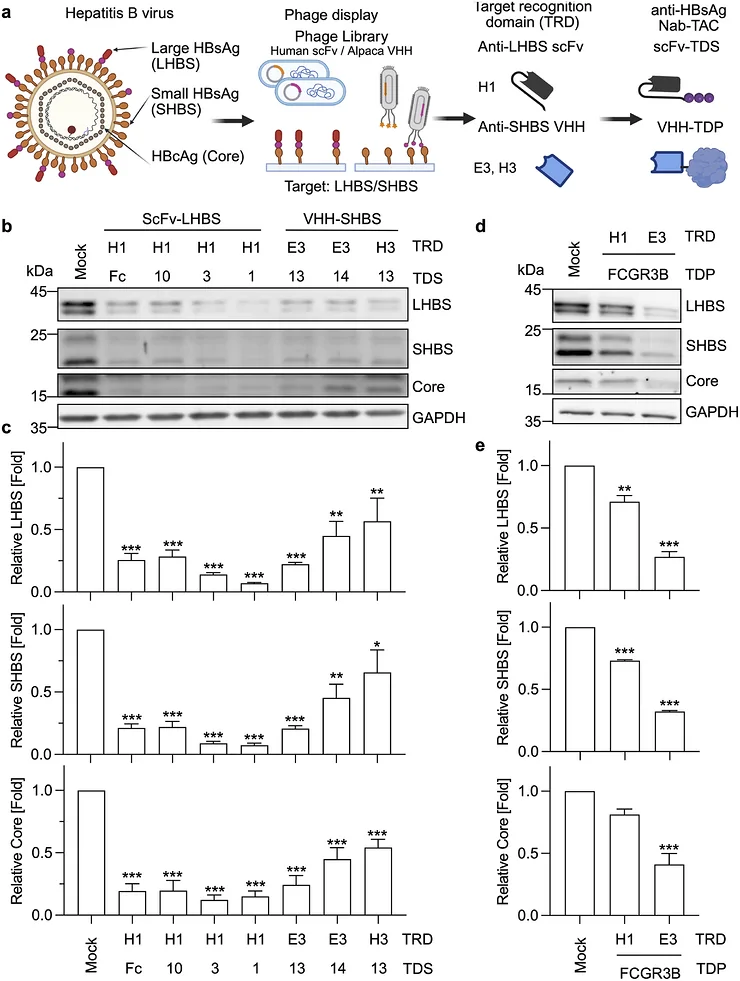
In vitro validation and functional screening of anti‐HBsAg Nab‐TAC constructs targeting HBV surface antigens. (a) Schematic illustration of HBV viral proteins, including large (LHBS), medium, and small (SHBS) surface antigens, and core protein (HBcAg). Anti‐LHBS single‐chain variable fragments (scFvs) were isolated from a human phage display library, whereas anti‐SHBS variable domain of heavy‐chain antibody (VHH) nanobodies were obtained from an alpaca VHH library. Identified target recognition domains (TRDs) were fused to TDS or TDP modules to generate bifunctional nanobody‐based targeted chimeras (Nab‐TACs). (b) Representative blots of LHBS, SHBS, and HBcAg levels following expression of representative TDS‐based Nab‐TAC constructs in HepG2 cells compared with mock control. (c) Densitometric quantification of Western blot signals shown in (b). Band intensities were normalized to the corresponding loading control and expressed as fold change relative to mock. Data are presented as mean ± SEM. (d) Representative blots of LHBS, SHBS, and HBcAg levels following expression of TDP‐based constructs (H1‐FCGR3B and E3‐FCGR3B) in HepG2 cells compared with mock control. (e) Densitometric quantification of Western blot signals shown in (d). Band intensities were normalized to the corresponding loading control and expressed as fold change relative to mock. Data are presented as mean ± SEM. Data are representative of at least two independent experiments. All datasets were derived from three independent biological replicates (n = 3). Data in (c) and (e) were analyzed using ordinary one‐way ANOVA followed by Dunnett's multiple comparisons test. Statistical significance is indicated as follows: ∗*p* < 0.05; ∗∗*p* < 0.01; ∗∗∗*p* < 0.001; ns, not significant. Statistical analyses were performed using GraphPad Prism 10.

Functional evaluation in the HBV replicon system showed that six Nab‐TAC candidates significantly reduced both SHBS and LHBS levels, as assessed by Western blot analysis (Figure 4b,c). Notably, surface antigen depletion was accompanied by concurrent reduction of HBV core antigen (HBcAg), suggesting that targeting HBsAg may influence viral protein homeostasis and particle assembly. Inhibitor‐based validation using H1‐TDS#10 showed that bafilomycin A1 treatment significantly rescued LHBS protein levels, confirming that H1‐TDS#10‐mediated degradation operates predominantly through a lysosome‐dependent pathway (Figure S3a,b).

For TDP‐based constructs, FCGR3B was selected based on its TRD‐independent concordant degradation activity across all screening systems (Figures 2a,e and 3b) and proteasome‐dependent mechanism confirmed by MG132 rescue (Figure S2). FCGR3B was combined with scFv H1 and VHH E3 to generate H1‐FCGR3B and E3‐FCGR3B constructs. Among all Nab‐TAC candidates evaluated, E3‐FCGR3B demonstrated the most pronounced reduction of HBV viral proteins (Figure 4d,e), highlighting FCGR3B as a highly effective degradation effector when coupled to nanobody‐mediated target recognition.

### 2.5 In Vivo Validation of Nab‐TAC‐mediated HBsAg Clearance

Among the validated degradation modules, E3‐FCGR3B was prioritized for in vivo validation based on its concordant and significant degradation activity across all cellular contexts and screening platforms (Figures 2a,e and 3b; Tables S5‐S6, S9‐S10, S15‐S16), proteasome‐dependent mechanism confirmed by MG132 rescue (Figure S2), and its activity independent of subcellular localization. To evaluate in vivo antiviral efficacy, E3‐FCGR3B was administered in a hydrodynamic injection mouse model of HBV, with assessments at day 2 and 7 post‐injection (Figure 5a). Immunohistochemical analysis of liver sections revealed a significant reduction in viral surface (LHBS and SHBS) and core antigens at both time points in the E3‐FCGR3B treated group compared with controls (Figure 5b,c). Western blot analysis confirmed substantial decreases in intracellular viral antigens (Figure 5d,e). Serologically, serum HBsAg levels were significantly reduced at both day 2 and 7 (Figure 5f), whereas HBeAg levels decreased significantly at day 2 but not at day 7, suggesting differential antigen clearance kinetics in vivo. At day 7, serum HBV DNA was also significantly reduced in the E3‐FCGR3B‐treated group (Figure S4a), indicating that Nab‐TAC‐mediated antigen degradation is associated with suppression of systemic viral burden.

**FIGURE 5:**
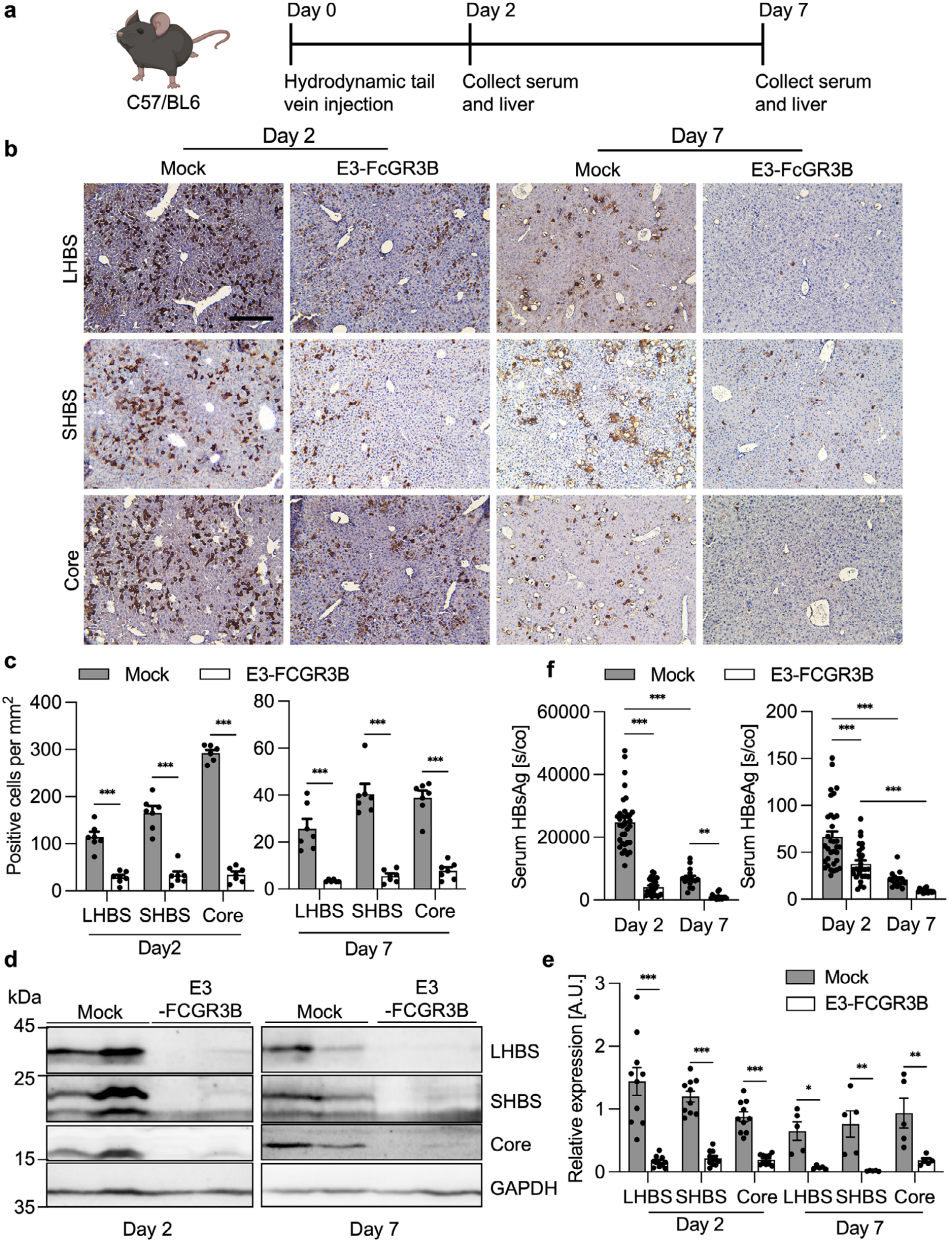
In vivo validation of anti‐HBsAg Nab‐TAC E3‐FCGR3B in the HBV hydrodynamic injection mouse model. (a) Schematic representation of the hydrodynamic HBV transfection mouse model. Mice received tail‐vein hydrodynamic injection of HBV replicon vector (pHBV3.6) together with either pcDNA3.1 vector control or pcDNA3.1‐E3‐FCGR3B construct on Day 0. Serum and liver tissues were collected at Day 2 and Day 7 for virological and biochemical analyses. (b) Representative immunohistochemical (IHC) staining of liver sections showing hepatic expression of LHBS, SHBS, and HBcAg in mice co‐injected with pHBV3.6 and pcDNA3.1 vector control or pcDNA3.1‐E3‐FCGR3B. Marked reduction of surface antigen‐positive hepatocytes was observed at both Day 2 and Day 7, indicating sustained in vivo degradation activity. Scale bar, 0.25 mm. (c) Quantification of IHC‐positive cells per mm^2^ corresponding to (b). For each mouse, two random fields were analyzed. Data are presented as mean ± SEM (n = 3 mice per group). (d) Representative blots of hepatic LHBS, SHBS, and HBcAg protein levels in mice receiving E3‐FCGR3B compared with vector control at Day 2 and Day 7. (e) Densitometric quantification of Western blot signals shown in (d). Band intensities were normalized to the corresponding loading control and expressed as relative expression (arbitrary units, A.U.). Data are presented as mean ± SEM (Day 2., n = 10; Day 7., n = 5). (f) Serum HBsAg and hepatitis B e antigen (HBeAg) levels measured at Day 2 and Day 7. in vector control and E3‐FCGR3B‐treated groups, demonstrating systemic reduction of circulating viral antigens. Data are presented as mean ± SEM (Day 2., n = 33; Day 7., n = 15). Data are representative of at least two independent experiments. Data in (c) and (e) were analyzed using two‐way ANOVA followed by Šídák's multiple comparisons test. Data in (f) were analyzed using two‐way ANOVA followed by Fisher's LSD multiple comparisons test. Statistical significance is indicated as follows: ∗*p* < 0.05; ∗∗*p* < 0.01; ∗∗∗*p* < 0.001. Statistical analyses were performed using GraphPad Prism 10.

To assess the safety profile of Nab‐TAC, we monitored hepatotoxicity markers in vivo. Serum aspartate aminotransferase (AST) and alanine aminotransferase (ALT) levels showed no significant differences between E3‐FCGR3B‐ and mock‐treated groups at either day 2 or day 7 (Figure S4b–e), confirming that Nab‐TAC treatment does not exacerbate liver injury under the conditions tested. Body weight was monitored daily throughout the study, with no significant deviations observed in the E3‐FCGR3B group compared with controls (Figure S4f,g), further supporting the absence of acute toxicity. For comparison, a single oral dose of tenofovir alafenamide (TAF) administered at day 0 produced no detectable reduction in intrahepatic HBV proteins or serum HBsAg at day 2 (Figure S5a,b), highlighting the mechanistic distinction between Nab‐TAC and current nucleos(t)ide‐based antivirals. Collectively, these results demonstrate that E3‐FCGR3B Nab‐TAC achieves robust intrahepatic degradation of HBV surface antigens, reduces systemic viral DNA, and does not induce detectable hepatotoxicity, supporting its potential as a therapeutic strategy complementary to existing antiviral treatments.

## 3 Discussion

### 3.1 Rationale and Modular Design of the Nab‐TAC Platform

Targeted protein degradation has transformed drug discovery for “undruggable” intracellular targets, yet most validated platforms, including PROTACs, molecular glues, AUTACs, and ATTECs, depend on small‐molecule ligands that are difficult to develop against viral proteins lacking deep, well‐defined binding pockets [43, 44, 45]. Antibody‐based degraders circumvent this limitation by exploiting the high specificity of paratope‐driven recognition, but existing formats are constrained in scope. For example, the Trim‐Away mechanism requires high‐endogenous TRIM21 expression and is incompatible with cells in which TRIM21 is limiting [46]; AbTAC and PROTAB are restricted to cell‐surface targets via RNF43/ZNRF3 recruitment [47]; LYTAC and AUTAC act predominantly on extracellular or membrane substrates through lysosomal routing [45]. None of these platforms readily accesses cytosolic, ER‐resident, or membrane‐embedded viral proteins such as HBsAg.

Nab‐TAC was designed to fill this gap. By coupling a single‐domain nanobody to a compact target degradation signal, this platform retains intracellular folding stability, accepts swap‐in nanobodies against virtually any antigen, and is small enough for plasmid, mRNA, or AAV delivery. The 14‐member TDS library spans ubiquitin‐proteasome, autophagy, lysosomal, and chaperone‐mediated routes, so a single nanobody can be paired with effectors of distinct mechanisms in a parallel, head‐to‐head fashion. This modularity, combined with cytosolic accessibility and proteome‐wide flexibility, distinguishes Nab‐TAC from the antibody‐degrader formats described above and positions it as a generalizable platform for clearing intracellular viral proteins. The same architecture should, in principle, extend to other persistent viral infections that depend on intracellular accessory proteins, such as Epstein–Barr virus LMP1, human papilloma virus E6/E7, or HIV Nef proteins [48], provided that high‐affinity nanobodies can be raised against the relevant cytosolic epitopes.

### 3.2 Mechanistic Differences Across NanoBiT and vhhGFP Platforms

A central methodological contribution of this study is the NanoBiT‐based quantitative screening platform [21], which converts proximity‐induced degradation into a luminescence readout amenable to medium‐throughput benchmarking. In a two‐plasmid transient co‐transfection system, stochastic plasmid uptake produces three cell populations: reporter‐only cells, effector‐only cells, and doubly transfected cells, where proximity‐induced degradation is active exclusively in the last population. NanoBiT luminescence requires simultaneous intracellular co‐expression of LgBiT and HiBiT‐TDP to reconstitute an active NanoLuc enzyme, so the signal arises only from cells in which proximity‐induced degradation can occur. As a high‐affinity, enzymatic readout with a wide dynamic range, NanoBiT resolves changes in this functionally active population with high sensitivity and is therefore the most appropriate platform for primary high‐throughput screening. In contrast, Western blot integrates LgBiT signals from both reporter‐only and doubly transfected cells, inflating the total LgBiT signal and attenuating the apparent reduction relative to luminescence. The vhhGFP system shares this limitation, but with an additional caveat: reporter‐only cells remain fluorescence‐positive even without the vhh‐effector, because EGFP fluorescence is intrinsic and requires no second component. Consequently, both Western blot and vhhGFP fluorescence underestimate true degradation efficiency relative to NanoBiT, which remains the most specific readout as it is completely blind to non‐degrading cells. Together, these findings establish that NanoBiT and vhhGFP are complementary rather than redundant: NanoBiT provides high‐sensitivity primary screening, while vhhGFP serves as orthogonal confirmation for geometrically sensitive candidates, and Western blot provides definitive protein‐level validation of selected hits. A systematic comparison of the three platforms, including their sensitivity, throughput, detection time, specificity, and key limitations, is provided in Table S19.

Among the nine TDP modules tested, FCGR3B unexpectedly emerged as a potent effector in both HepG2 and HeLa contexts, despite its physiological role as a glycosylphosphatidylinositol (GPI)‐anchored Fcγ receptor on neutrophils [41, 49]. GPI signal peptide cleavage is required for FCGR3B‐dependent degradation and the cleaved tail of GPI‐anchored proteins is a potent trans‐acting degron, possibly related to normal quality control of GPI‐anchored proteins [50]. The identification of this noncanonical, trans‐acting degron mechanism through split‐luciferase complementation‐based screening thus expands the conceptual and functional landscape of targeted protein degradation beyond canonical E3 ligase recruitment. Another notable TDP is PRR20A, whose vhhGFP configuration showed a paradoxical increase in EGFP fluorescence, concordant with Western blot (Figure 2e–g, Tables S9–S11), indicating a genuine increase in protein abundance rather than a readout artifact. We attribute this to a hook‐effect‐like behavior, where excess effector overexpression may saturate components of the ubiquitin‐proteasome system, analogous to the PROTAC hook effect in which excess heterobifunctional occupancy favors non‐productive binary complexes over productive ternary degradation complexes [51]. In this context, NanoBiT luminescence remained a reliable indicator of degradation precisely because the HiBiT‐TDP configuration is less susceptible to this expression‐dependent confounder. Both readouts faithfully reported protein abundance in their respective configurations, and the PRR20A case illustrates that discordance between platforms reflects configuration‐specific behavior rather than failure of either system.

The discordance between luminescence and fluorescence readouts observed for a subset of TDP candidates is mechanistically attributable to steric hindrance imposed by specific TDP modules on the HiBiT tag, reducing its complementation efficiency with LgBiT independent of actual protein degradation. UBE2B, GABARAP, EID1, and PRR20A showed significant luminescence reduction without a corresponding fluorescence decrease in the HiBiT‐TDP configuration (Figure 3b, Tables S15–S16); however, when re‐evaluated as vhhGFP‐TDP fusions in the same stable HeLa LgBiT‐EGFP‐NLS cell line, all four candidates recovered significant fluorescence reduction (Figure 3e, Table S18), confirming that their degradation capacity is intact and that the discordance reflects TRD size‐dependent steric interference rather than absence of degradation activity. FCGR3B, the only candidate evaluated across all assay systems, showed concordant degradation activity in HiBiT‐TDP luminescence, vhhGFP‐TDP fluorescence, and western blotting, supporting the validity of its selection and downstream in vitro and in vivo validation (Figures 4 and 5).

A broader principle emerging from these observations is that platform selection in proximity‐based degradation assays should be guided by effector size. Compact, peptide‐based degrons such as TDS are well suited to the NanoBiT assay, which depends on close HiBiT‐LgBiT proximity for complementation, whereas larger protein‐based effectors such as full‐length TDP modules are better suited to complementation‐independent fluorescence platforms such as vhhGFP, which are less susceptible to steric interference. Nonetheless, NanoBiT performed effectively for most TDP candidates in this study, with only a subset (UBE2B, GABARAP, EID1, and PRR20A) showing complementation interference that was resolved by vhhGFP confirmation. These cases define two functional classes, TRD size‐sensitive (UBE2B, GABARAP, EID1, PRR20A) and TRD‐independent (FCGR3B, SPOP, LY6D) and highlight that effector size is a guiding principle rather than a strict rule. Cross‐platform validation remains essential for confidently identifying true degradation effectors.

In this study, the cell‐line‐dependent activity observed across TDS candidates reflects a core principle in targeted protein degradation that degrader efficacy is largely dictated by the intracellular abundance of the recruited E3 ligase or autophagy receptor [40, 52]. For example, TRIM7 (TDS #5‐#8) is highly expressed in HeLa but nearly absent in HepG2, whereas Keap1 (TDS #2) and Nix/BNIP3L (TDS #9) show the opposite pattern [53, 54], consistent with their respective cell‐line‐preferential activities. Beyond E3 ligase availability, TDP degradation efficiency was generally higher in HeLa than in HepG2 cells (Figure 2a,e), which we attribute to intrinsic differences in chaperone abundance, proteasome activity, and ER quality‐control set points between the two cell lines. These observations should be interpreted as mechanistically informative features of the screening data rather than technical inconsistencies. This context dependence further underscores the value of multi‐cell‐line evaluation, as activity in a single cell type would be insufficient to predict degradation competence in therapeutically relevant contexts, such as hepatocytes.

### 3.3 Functional Clearance of HBV Antigens and Coordinated Capsid Reduction

In this study, the lead construct E3‐FCGR3B efficiently reduced intracellular HBsAg in transiently transfected HepG2 cells (Figure 4d,e) and significantly diminished both intrahepatic and serum HBsAg in the HBV hydrodynamic injection mouse model (Figure 5). HBsAg comprises three co‐translated isoforms, LHBS, MHBS, and SHBS, all of which share the S domain as a common structural component. The E3 nanobody targets this shared S domain, thereby enabling simultaneous degradation of all three isoforms. Since HBsAg is the principal structural component of subviral particles, which outnumber infectious virions by 10^3^ to 10^5^‐fold and constitute the dominant form of circulating HBsAg in chronic infection [2], E3‐FCGR3B‐mediated degradation of HBsAg is expected to reduce not only intracellular viral antigen but also subviral particles, potentially alleviating the antigen‐driven immune exhaustion that underlies HBV persistence.

A noteworthy finding is the parallel reduction of viral core antigen, which was not the direct target of Nab‐TAC in this study. Several non‐exclusive mechanisms may account for this observation. First, acute depletion of envelope proteins disrupts virion assembly and traps nucleocapsids in the cytosol, where they are susceptible to clearance by basal autophagy [55]. Second, loss of HBsAg may relieve feedback signals that stabilize cccDNA‐driven transcription and subsequent translation, thereby lowering core antigen synthesis [56]. Third, we propose an additional mechanism: during intracellular virion morphogenesis, nucleocapsids are recruited to envelope protein‐enriched membrane compartments through direct core‐LHBs interaction prior to budding [57]. Degradation of HBsAg by E3‐FCGR3B at this pre‐budding stage may therefore co‐eliminate envelope‐associated nucleocapsids before virion secretion, effectively targeting both components simultaneously within the same intracellular compartment. Together, these observations suggest that Nab‐TAC exerts antiviral effects beyond its primary target, perturbing multiple steps of the HBV life cycle in a manner that current antiviral therapies cannot replicate.

### 3.4 Positioning Within the HBV Therapeutic Landscape

Here, we emphasize that Nab‐TAC complements rather than replaces existing therapies, and its mechanistic niche is distinct. In this study, the nucleos(t)ide analog TAF demonstrated effective HBV DNA suppression without a measurable reduction in HBsAg (Figure S4), recapitulating clinical experience and highlighting the unmet need addressed by Nab‐TAC [58, 59]. While RNA‐targeting agents acting at the mRNA level, including the antisense oligonucleotide bepirovirsen and the siRNA agents JNJ‐3989 and AB‐729, have produced sustained HBsAg loss in a subset of treated participants in recent Phase 2 trials [60, 61], these modalities may reduce new HBsAg synthesis without directly clearing the existing intracellular protein pool. Nab‐TAC operates downstream at the post‐translational level, degrading HBsAg already present within hepatocytes, and therefore offers a mechanistically orthogonal action that may synergize with upstream mRNA knock‐down strategies to more completely eliminate both newly synthesized and pre‐existing antigen.

A rational combination roadmap would therefore pair Nab‐TAC with NAs (to suppress replication), ASO/siRNA (to limit new HBsAg production), and immunomodulators or therapeutic vaccines (to restore adaptive immunity). Such multi‐pronged regimens are increasingly recognized as necessary to achieve functional cure of chronic hepatitis B [62, 63], and Nab‐TAC provides the missing post‐translational clearance arm. Taken together, Nab‐TAC introduces a programmable, mechanism‐orthogonal modality for intracellular viral protein degradation, validated here against HBsAg in both cellular and in vivo HBV models, and provides a foundation for next‐generation antiviral and protein‐degrader therapeutics.

### 3.5 Limitations of the Study

Despite these promising results, several limitations should be acknowledged. First, while initial evidence supports involvement of the lysosomal pathway in TDS‐based constructs, the relative contributions and mechanistic interplay between lysosomal and proteasomal degradation require further elucidation. Second, antigen suppression was evaluated in a hydrodynamic injection mouse model, which reflects transient antigen expression rather than chronic HBV infection sustained by cccDNA or integrated viral DNA; the durability and breadth of Nab‐TAC efficacy therefore remain to be validated in more physiologically relevant models of chronic infection, such as humanized liver chimeric mice or woodchuck models. Third, a shared limitation of both the NanoBiT and vhhGFP assays is that transient two‐plasmid co‐transfection generates reporter‐only cells that contribute undegraded LgBiT or EGFP signal to bulk readouts without participating in degradation, causing both platforms to underestimate true degradation efficiency. Finally, current findings rely on plasmid‐based intracellular expression, and clinically relevant hepatocyte delivery modalities, such as lipid nanoparticles or adeno‐associated viral vectors, represent a key translational challenge that warrants dedicated investigation. Addressing these limitations will be essential for advancing Nab‐TAC toward clinical development.

## 4 Materials and Methods

### 4.1 Plasmids and Molecular Cloning

The coding sequences of LgBiT and HiBiT were obtained from Promega and subcloned into pcDNA3.1 (Invitrogen) using In‐Fusion HD cloning (Takara Bio, 638943). The vhhGFP nanobody sequence (Addgene #136619) was cloned into pcDNA3.1 to generate pcDNA3.1‐vhhGFP [64, 65]. For degradation module screening, fourteen targeted degradation signal/TDS candidates were fused downstream of HiBiT to generate HiBiT‐TDS constructs. Corresponding constructs were also generated in the pcDNA3.1‐vhhGFP‐HiBiT backbone. Eight targeted degradation protein/TDP candidates (Sino Biological) were cloned into both HiBiT and vhhGFP‐HiBiT vectors to generate HiBiT‐TDP and vhhGFP‐HiBiT‐TDP constructs. EGFP was fused downstream of HiBiT (HiBiT‐EGFP) as a non‐degrading control. For antigen‐targeting constructs, anti‐SHBS nanobodies (vhhSHBS) and anti‐LHBS scFvs (scFvLHBS) were cloned into pcDNA3.1 and fused in‐frame with selected TDS or TDP modules. For nuclear degradation assays, pYA5‐PiggyBac_ITR‐LgBiT‐EGFP‐NLS was constructed by fusing EGFP and a nuclear localization signal to LgBiT. The HBV replicon plasmid pHBV3.6 was provided by Dr. Pei‐Jer Chen (National Taiwan University, Taiwan) [66]. PiggyBac vectors were provided by Dr. Masato Kanemaki (National Institute of Genetics, Japan). The vhhGFP vector was a gift from Kai Johnsson (Addgene plasmid # 136619) [65]. All constructs were verified by Sanger sequencing.

### 4.2 Cell Culture

HeLa (RRID:CVCL_0030), HepG2 (RRID:CVCL_0027), HeLaLgBiT‐EGFP‐NLS reporter cell lines were maintained in DMEM supplemented with 10% FBS and 1% penicillin‐streptomycin. A monoclonal HeLa cell line stably expressing LgBiT‐EGFP‐NLS was established by sorting single eGFP‐positive cells after transfection with pYA5‐PiggyBac_ITR‐LgBiT‐EGFP‐NLS and pCMV‐PiggyBac transposase at a 5:1 ratio. Clonal populations were expanded and screened for stable and homogeneous nuclear eGFP expression by fluorescence microscopy. A clone exhibiting robust and uniform nuclear LgBiT‐EGFP‐NLS expression was selected and used for subsequent nuclear protein degradation assays.

### 4.3 NanoBiT Selection Assay

Hela, HepG2, and Hela‐LgBiT‐EGFP‐NLS were seeded into 96‐well white plates at approximately 70% confluency for overnight and transfected with of pcDNA3.1‐LgBiT and pcDNA3.1‐HiBiT‐TDS or pcDNA3.1‐HIBIT‐TDP at the molar ratio of 1:5 with GenJet In Vitro DNA Transfection Reagent (SignaGen, MD, USA. Cat. #SL100489) at a DNA‐to‐reagent ratio of 1:3 (w/v; 100 ng total DNA with 0.3 µL reagent per well) according to the manufacturer's instructions. After 24‐ to 72‐h post‐transfection, the culture medium in the plate was aspirated, washed with PBS for 1 time and treated with 100 µL OPTI‐MEM reaction mixture containing 0.5 µL of Nano‐Glo luciferase assay substrate, and 9.5 µL of luciferase assay diluent (Promega, WI, USA. Cat.# N1110) was added into each well. The luminescence signal was recorded every 2 min continuously for 30 min using a microplate reader (BioTek Synergy HTX, VT, USA) at 37°C with a time‐lapsed kinetics program. To calculate LgBiT reduction by HiBiT candidates, luminescence data from the timepoint showing the highest signal in the negative control sample was selected for downstream calculation. Reduction (%) = [1‐(luminescence signal of test sample)/ (luminescence signal of vehicle control sample)] × 100.

### 4.4 VhhGFP Degradation Assay

HeLa, HepG2, and HeLa‐LgBiT‐EGFP‐NLS were seeded into 96 well black plates at approximately 70% confluency for overnight and co‐transfected with pcDNA3.1‐LgBiT /pcDNA3.1‐HiBiT‐TDS or pcDNA3.1‐HIBIT‐TDP at the molar ratio of 1:5 with GenJet In Vitro DNA Transfection Reagent (SignaGen) at a DNA‐to‐reagent ratio of 1:3 (w/v; 100 ng total DNA with 0.3 µL reagent per well) according to the manufacturer's instructions. After 72‐h post‐transfection, the culture medium in the plate was aspirated, washed with PBS for 1 time and replaced with 100 µL PBS into each well. The fluorescence signal was recorded using a microplate reader (BioTek Synergy HTX, VT) at 37°C with an endpoint program. To calculate EGFP reduction by vhhGFP candidates, fluorescence data from the endpoint was selected for downstream calculation. Reduction (%) = [1‐(fluorescence signal of test sample)/ (fluorescence signal of vehicle control sample)] × 100.

### 4.5 Functional Screening of Anti‐HBsAg Nab‐TAC Constructs

To validate and screen the potential anti‐HBsAg Nab‐TAC constructs, HepG2 cells were seeded in 6‐cm dishes at approximately 70% confluency and transfected with pHBV3.6 and Nab‐TAC plasmids at a molar ratio of 1:5 ratio using GeneJET transfection reagent, (2.4 µg total DNA and 7.2 µL GenJet reagent, DNA‐to‐reagent ratio of 1:3, w/v) according to the manufacturer's instructions. Seventy‐two hours post‐transfection, cells were collected for the following Western blotting analysis.

### 4.6 Western Blotting Analysis

For Western blot analysis, cells were seeded into 6‐cm dishes at approximately 70% confluency and transfected with 2.4 µg total DNA and 7.2 µL GenJet reagent (DNA‐to‐reagent ratio of 1:3, w/v) under otherwise identical conditions. To detect the various protein levels in cell lines, the cells were harvested by trypsin and lysed in RIPA buffer (1% NP‐40, 0.1% SDS, 0.5% sodium deoxycholate, 150 mM NaCl, 50 mM Tris‐HCl, pH 7.4) supplemented with 1X protease inhibitor cocktail (Roche, IN, USA. Cat. #4693116001). The lysing samples were incubated on ice for 30 min and scraped every 10 min, followed by 13000 rpm centrifugation at 4°C for 15 min. The supernatants were collected for protein quantification by Bradford assay, by which the protein concentrations were determined with spectrophotometer measurements. The protein samples were mixed with a 2X Laemmli sample buffer containing β‐Me (65.8 mM Tris‐HCl, pH 6.8, 2.1% SDS, 26.3% (w/v) glycerol, 0.01% bromophenol blue. Before use, add 5% β‐mercaptoethanol) and then incubated at 100°C for 15 min before loading to 10% SDS‐PAGE gels. A total of 30 µg cell lysates were loaded per each lane. After electrophoresis, the samples were transferred to the PVDF membrane (Millipore, MA, USA. Cat. #IPVH00010) by 100 V for 1 hr. The membrane was incubated with primary antibodies (Anti‐HBV pres1 monoclonal antibody, clone S1‐7H11, 1:1000, Creative diagnostics, NY, USA. Cat. #DMAB3518; Anti‐HBV S monoclonal antibody, clone HB1, 1:1000, a gift from Dr. Aurelija Žvirblienė (Vilnius University, Lithuania); Anti‐HBV Core monoclonal antibody, clone LTK, 1:3000, a gift from Dr. Hui Lin Wu (National Taiwan University, Taiwan); anti‐GAPDH monoclonal antibody, 1:5000, Genetex, CA, USA. Cat. #GTX100118, RRID: AB_1080976; Anti‐beta Tubulin polyclonal antibody, 1:5000, Novus Biologicals, CO, USA. Cat. #NB600‐936, RRID: AB_10000656; Anti‐LgBiT monoclonal antibody, 1:2000, Promega, Cat. #N710A) at 4°C overnight. After washing three times with phosphate buffer saline with 0.05% Tween 20 (PBST) every 10 min, the PVDF membrane was then incubated with anti‐mouse horseradish peroxidase (HRP)‐conjugated secondary antibodies (Jackson ImmunoResearch, PA, USA. Cat. #115‐035‐003, RRID: AB_10015289) or anti‐rabbit HRP‐conjugated secondary antibody (Jackson ImmunoResearch, Cat. #111‐035‐003, RRID: AB_2313567) for 30 min at room temperature. Finally, protein bands on washed PVDF membrane were visualized using modified ECL reagent (ddH_2_O, 100 nM Tris‐HCl pH8.0, 200 µM p‐coumaric acid, 1.25 mM luminal, 0.001%H_2_O_2_) and detected by ImageQuant LAS 4000 digital imaging system (GE Healthcare, MA, USA) within the detection linear range of the system. The images were quantified by ImageQuantTL (GE Healthcare).

### 4.7 Immunofluorescence Imaging

Cells grown on coverslips were washed with PBS and then incubated with PTEMF fixation solution (20 mM PIPES pH 6.8, 0.2% Triton X‐100 10 mM EGTA, and 4% formaldehyde) at room temperature for 15 min. The fixation buffer was aspirated and replaced with PBST at room temperature for 10 min. The coverslips were then washed with PBST and incubated with 4',6‐diamidino‐2‐phenylindole (DAPI) for 30 min. Finally, the coverslips were washed another three times (2, 2, and 5 min) with PBST, rinsed with ddH2O once, and mounted with VECTASHIELD Antifade Mounting Medium (Vector, CA, USA. Cat. #H‐1000‐10). Images were acquired with corresponding laser wavelengths by Leica DMI6000 microscope with an HCX PL FL 20X/0.4 objective lens and Andor Neo sCMOS camera, which were all processed by MetaMorph software (Molecular Devices, LLC. San Jose, CA, USA).

### 4.8 Nanobody Discovery by Phage Display

Anti‐SHBS nanobodies were identified from a naïve alpaca VHH phage display library (Elabscience, Hubei, China) using a standard M13K07 helper phage‐based biopanning strategy. The glycerol‐preserved VHH library in *E. coli* TG1 was expanded in 2YT medium containing ampicillin and 2% glucose at 37°C (220 rpm) to mid‐log phase (OD600 = 0.4‐0.6). M13K07 helper phage was added at a 20:1 ratio (phage:bacteria) and incubated for 30 min without shaking followed by 1 h with shaking at 37°C. Cells were pelleted and resuspended in 2YT supplemented with ampicillin and kanamycin and cultured overnight at 30°C to produce phage particles. Phages were purified from supernatants by two rounds of PEG‐NaCl precipitation and resuspended in PBS. Library titer was determined by TG1 infection (approximately 3 × 10^1^ ^3^ pfu mL^−^^1^).

For biopanning, recombinant SHBS antigen (20 µg) was coated onto immunotubes at 37°C for 2 h and blocked with 2% milk in PBS. Approximately 3 × 10^1^ ^2^ pfu of phage library diluted in blocking buffer were incubated with immobilized antigen for 2 h at 37°C. After extensive washing with 0.05% PBST (stringency increased over four rounds), bound phages were eluted using glycine‐HCl (pH 2.0) and immediately neutralized with Tris‐HCl (pH 9.5). Eluted phages were amplified in TG1 cells with M13K07 rescue and purified by PEG precipitation before subsequent panning rounds. Four rounds of selection were performed to enrich antigen‐specific binders. Individual clones from the fourth round were rescued with helper phage, and phage‐containing supernatants were subjected to monoclonal phage ELISA. SHBS antigen and unrelated control protein (100 ng per well) were coated onto ELISA plates, blocked with 2% milk in PBS, and incubated with phage supernatants. Bound phages were detected using HRP‐conjugated anti‐M13 antibody and TMB substrate, and absorbance was measured at 450 nm. Positive clones (B3, C3, E3, F3, G3, and H3) exhibiting strong SHBS‐specific binding with minimal background were sequenced and subcloned for downstream functional validation.

### 4.9 ScFv Discovery by Phage Display

Anti‐LHBS single‐chain variable fragments (scFvs) were isolated from a human antibody phage display library generously provided by Dr. Han‐Chung Wu [67, 68]. Library amplification, helper phage rescue, PEG precipitation, and titer determination were performed as described above for the VHH library. Individual clones from the final round were screened by monoclonal phage ELISA against LHBS antigen and unrelated control protein. Positive clones C10‐2 and H1 showing specific binding were sequenced and subcloned into pcDNA3.1 for fusion with TDS or TDP modules.

### 4.10 HBV Hydrodynamic Injection Mouse Model

The HBV hydrodynamic injection model was established as previously described [69]. Briefly, 6‐8‐week‐old C57BL/6 (RRID: IMSR_JAX:000664) mice were intravenously injected via the tail vein with 5 µg of pHBV3.6 plasmid and 25 µg of pcDNA3.1‐E3‐FCGR3B or 25 µg of vector control diluted in sterile PBS at a volume equivalent to 8–10% of body weight within 5–8 s. Mice were sacrificed at Day2 and Day7. Serum and liver tissues were collected for downstream analyses, including antigen quantification, Western blotting, and immunohistochemistry. All animal experiments were performed in accordance with the guidelines of the Institutional Animal Care and Use Committee of National Tsing Hua University (IACUC‐11312H099).

### 4.11 Immunohistochemistry Staining

Mouse liver tissues were fixed in 10% neutral‐buffered formalin for 24 h, embedded in paraffin, and sectioned at 5 µm thickness. Sections were deparaffinized in xylene, rehydrated through graded ethanol, and subjected to heat‐induced antigen retrieval in citrate buffer (pH 6.0). Endogenous peroxidase activity was quenched using hydrogen peroxide solution. Sections were incubated with primary antibodies against SHBS, LHBS, and HBV core for 1 h at room temperature. Detection was performed using the Abcam Mouse and Rabbit Specific HRP/DAB Detection IHC Kit (Abcam, Cambridge, UK. Cat. #ab64264) according to the manufacturer's instructions. DAB was used as chromogen and hematoxylin for nuclear counterstaining. Slides were imaged using a bright‐field microscope, and staining intensity was quantified using ImageJ software.

### 4.12 Extracellular Serum Viral Antigen Quantification

Serum HBsAg and HBeAg levels were measured using the ARCHITECT HBsAg Qualitative II and HBeAg Qualitative assays (Abbott Diagnostics, IL, USA) according to the manufacturer's protocols. Assays were performed on the ARCHITECT i System (Abbott Diagnostics). Results were expressed as signal‐to‐cutoff (S/CO) ratios. Samples with S/CO ≥1.0 were considered reactive. Quantitative comparisons between groups were performed using normalized S/CO values.

### 4.13 Quantification and Statistical Analysis

All data are presented as mean ± standard deviation (SD) unless otherwise indicated. Statistical analyses were performed using GraphPad Prism 10 software. Comparisons among multiple groups were conducted using one‐way ANOVA or two‐way ANOVA followed by appropriate post hoc testing. For multiple group comparisons, variance homogeneity was first assessed using the Brown–Forsythe test. When the Brown–Forsythe test yielded p > 0.05 (confirming homogeneity of variances), ordinary one‐way ANOVA was applied; when p ≤ 0.05 (indicating heterogeneity of variances), Brown–Forsythe and Welch's ANOVA was applied instead. Two‐group comparisons were performed using an unpaired two‐tailed Student's t‐test. A p‐value <0.05 was considered statistically significant. The sample sizes (n) are provided in the figure legends.

## Author Contributions

**Chih‐Chien Chiu**: validation, visualization. **Richard Kuan‐Lin Lee**: resources, methodology, investigation, validation. **Max Yu‐Chen Pan**: conceptualization, investigation, writing – original draft, validation, methodology, formal analysis, project administration. **Xin‐Yee Chin**: investigation, validation, visualization. **Hong Vinh Nguyen**: validation, visualization. **Ting‐Hui Lee**: formal analysis, visualization, validation, investigation. **Eva Yi‐Hsuan Wu**: conceptualization, investigation, validation, methodology. **Ming‐Hong Chao**: validation, visualization. **Tzu‐Ning Peng**: validation, visualization, investigation. **Lily Hui‐Ching Wang**: conceptualization, methodology, supervision, resources, funding acquisition, investigation, writing – review and editing, writing – original draft. **Han‐Chung Wu**: methodology, conceptualization, resources. **Yu‐Ting Chiu**: validation, visualization. **Yuan‐Shao Pao**: validation, visualization.

## Conflicts of Interest

The authors declare no conflicts of interest.

## Supporting information




**Supporting File**: advs76687‐sup‐0001‐SuppMat.docx.

## Data Availability

The data that support the findings of this study are available from the corresponding author upon reasonable request.
