# Supplementary material for: Programmable Nanobody‐Targeting Chimeras Enable Intracellular Viral Protein Degradation
Source: Adv Sci (Weinh). 2026 Jul 17:e76687. Online ahead of print. doi: 10.1002/advs.76687 (PMC13379255; doi:10.1002/advs.76687)
Supplement: Supplementary file 1 — Supporting File: advs76687‐sup‐0001‐SuppMat.docx. [file ADVS-9999-e76687-s001.docx]

**Supporting information**

**Supplemental Method**

**NanoBiT inhibitor assay**

To investigate the potential degradation pathway triggered by FCGR3B, inhibitor assays were performed in HeLa cells. Briefly, HeLa cells were seeded into 96 well black plates at approximately 70% confluency for overnight and co-transfected with pcDNA3.1-LgBiT /pcDNA3.1-HiBiT-FCGR3B at the molar ratio of 1:5 with using GenJet™ In Vitro DNA Transfection Reagent (SignaGen) at a DNA-to-reagent ratio of 1:3 (w/v; 100 ng total DNA with 0.3 µL reagent per well), according to the manufacturer’s instructions. At 48-hour post-transfection, cells were treated with the indicated inhibitors for 6 h prior to harvest. The following inhibitors were used: MG132 (10 μM), Bafilomycin A1 (10 μM), Chloroquine (10 μM), and U1866A (10 ng μ/L). After treatment, the culture medium in the plate was aspirated, washed with PBS for 1 time and treated with 100 μL OPTI-MEM reaction mixture containing 0.5 μL of Nano-Glo luciferase assay substrate, and 9.5μL of luciferase assay diluent (Promega) was added into each well. The luminescence signal was recorded every 2 min continuously for 30 min using a microplate reader (BioTek Synergy HTX, VT) at 37°C with a time-lapsed kinetics program. To calculate LgBiT reduction by HiBiT candidates, luminescence data from the timepoint showing the highest signal in the negative control sample was selected for downstream calculation.

**EGFP inhibitor assay**

To investigate the potential degradation pathway triggered by FCGR3B, inhibitor assays were performed in Hela cells. Hela cells were seeded into 96 well black plates at approximately 70% confluency for overnight and co-transfected with pcDNA3.1-EGFP /pcDNA3.1-vhhGFP-FCGR3B at the molar ratio of 1:5 using GenJet™ In Vitro DNA Transfection Reagent (SignaGen) at a DNA-to-reagent ratio of 1:3 (w/v; 100 ng total DNA with 0.3 µL reagent per well), according to the manufacturer's instructions. After 48-hour post-transfection, cells were treated with the indicated inhibitors for 6 h prior to harvest. The following inhibitors were used: MG132 (10 μM), Bafilomycin A1 (10 μM), Chloroquine (10 μM), and U1866A (10 ng μ/L). After treatment, the culture medium in the plate was aspirated, washed with PBS for 1 time and replaced with 100 μL PBS into each well. The fluorescence signal was recorded using a microplate reader (BioTek Synergy HTX, VT) at 37°C with a endpoint program. To calculate EGFP reduction by vhhGFP candidates, fluorescence data from the endpoint was selected for downstream calculation.

For Western blot analysis, cells were seeded into 6-cm dishes at approximately 70% confluency and transfected with 2.4 µg total DNA and 7.2 µL GenJet™ reagent (DNA-to-reagent ratio of 1:3, w/v) under otherwise identical conditions.

**Inhibitor Assay**

To investigate the degradation pathways involved in candidate-mediated protein clearance, inhibitor assays were performed in HepG2 cells. Briefly, HepG2 cells were seeded in 6-cm dishes at approximately 70% confluency overnight prior to transfection. HepG2 cells were transfected with pHBV3.6 and Nab-TAC plasmids at a molar ratio of 1:5 (2.4 µg total DNA and 7.2 µL GenJet™ reagent, DNA-to-reagent ratio of 1:3, w/v), according to the manufacturer’s instructions. At 48 h post-transfection, cells were treated with the indicated inhibitors for 6 h prior to harvest. The following inhibitors were used: MG132 (10 μM), Bafilomycin A1 (10 μM), Chloroquine (10 μM), and U1866A (10 ng/μL). After treatment, cells were harvested and lysed, and protein levels were analyzed by western blotting.

**Quantification of serum AST and ALT**

To evaluate liver function, mouse blood samples were collected at the time of sacrifice via cardiac puncture. Whole blood was centrifuged at 16,000 × g for 3 min, and the supernatant was transferred to a new tube and centrifuged again under the same conditions to obtain clarified serum. Serum alanine aminotransferase (ALT) and aspartate aminotransferase (AST) activities were measured by an external clinical laboratory using commercially available diagnostic reagents (DiaSys Diagnostic Systems) according to the manufacturer’s instructions. The assays were performed using an optimized UV kinetic method based on the International Federation of Clinical Chemistry (IFCC) protocol, which quantifies enzymatic activity through NADH oxidation-coupled reactions monitored spectrophotometrically.

**Tenofovir alafenamide treatment in HBV hydrodynamic injection mouse model**

For antiviral validation in the HBV hydrodynamic injection (HDI) mouse model, mice were administered tenofovir prior to hydrodynamic injection. Tenofovir (Vemlidy) was dissolved in sterile water to prepare a working solution immediately before use. Mice received tenofovir at a single dose of 30 mg/kg body weight (mpk) via oral gavage. The drug was administered prior to hydrodynamic injection to evaluate its antiviral effect during the early stage of HBV expression. Liver tissues were harvested at the experimental endpoint and analyzed by western blotting to assess HBV viral protein expression.

**Serum HBV DNA quantification**

Mouse blood samples were collected at the time of sacrifice and centrifuged at 16,000 × g for 3 min, followed by a second centrifugation under the same conditions to obtain serum. To remove contaminating plasmid DNA, serum samples were treated with DNase I (New England Biolabs #M0303) at 37 °C for 40 min prior to nucleic acid extraction. Viral DNA was then purified using the High Pure Viral Nucleic Acid Kit (Roche #11858874001) according to the manufacturer’s protocol.

Quantitative PCR (qPCR) was performed to determine HBV DNA copy number. A pHBV3.6 plasmid was used as the standard template to generate a calibration curve for absolute quantification. The following primers targeting the HBsAg coding region were used: Forward: 5’-GTGTCTGCGGCGTTTTATCA-3’, Reverse: 5’-GACAAACGGGCAACATACCTT-3’. HBV DNA copy numbers were calculated by interpolation from the standard curve generated using serial dilutions of the pHBV3.6 plasmid. qPCR was performed using an AriaMx Realtime PCR System (Agilent Technologies). Reactions were performed using SensiFAST SYBR Lo-ROX Kit (Bioline #BIO-94005) according to the manufacturer’s protocol.

### **Quantification and Normalization of NanoBiT Luminescence and EGFP Fluorescence Signals**

Raw luminescence (RLU) and fluorescence (RFU) values were normalized to the respective within-batch control to account for inter-experimental variability in transfection efficiency and cell number. For each biological replicate, the raw signal of each experimental group was divided by the mean raw signal of the control group within the same experiment and multiplied by 100, yielding a normalized signal expressed as a percentage of control (control = 100%): Normalized signal (%) = (Raw signal of experimental group ÷ Mean raw signal of control group) × 100. Degradation efficiency was subsequently expressed as reduction percentage: Reduction (%) = 100 − Normalized signal (%). Positive reduction values indicate decreased protein abundance relative to control, whereas negative values indicate increased signal, suggesting protein stabilization or enhanced complementation efficiency. Mean and standard deviation (SD) were calculated from the three individually normalized replicate values for each condition.

**Supplemental tables**

Table S1. Quantitative luminescence data for HiBiT-TDS candidates in HeLa cells at 24 h (Figure 1f).

| HiBiT-TDS | Normalized luminescence (%) | Reduction (%) | p value vs control |
| --- | --- | --- | --- |
| - | 100 ± 5.5 | 0 |  |
| 1 | 67.0 ± 2.4 | 33.0 | 0.002 (**) |
| 2 | 217.3 ± 10.9 | -117.3 | <0.001 (***) |
| 3 | 59.7 ± 2.9 | 40.3 | <0.001 (***) |
| 4 | 71.1 ± 2.9 | 28.9 | 0.007 (**) |
| 5 | 99.0 ± 5.1 | 1.0 | >0.99 (ns) |
| 6 | 110.7 ± 7.0 | -10.7 | 0.76 (ns) |
| 7 | 95.7 ± 2.9 | 4.3 | >0.99 (ns) |
| 8 | 157.2 ± 7.1 | -57.2 | <0.001 (***) |
| 9 | 121.9 ± 5.3 | -21.9 | 0.07 (ns) |
| 10 | 74.0 ± 3.6 | 26.0 | 0.02 (*) |
| 11 | 80.2 ± 1.9 | 19.8 | 0.12 (ns) |
| 12 | 85.4 ± 8.9 | 14.6 | 0.4 (ns) |
| 13 | 54.6 ± 1.9 | 45.4 | <0.001 (***) |
| 14 | 68.1 ± 1.8 | 31.9 | 0.003 (**) |

Values represent normalized luminescence signal (%, mean ± SEM, n=3) relative to HiBiT/LgBiT control (100%). Reduction (%) = 100 − normalized signal. Statistical comparisons performed by ordinary one-way ANOVA with Dunnett's post-hoc test vs. control. In the "p value vs control" columns, the symbols shown in parentheses denote significance levels: *p < 0.05, **p < 0.01, ***p < 0.001; ns = not significant (p ≥ 0.05). SEM, standard error of the mean.

Table S2. Quantitative luminescence data for HiBiT-TDS candidates in HeLa cells at 48 h (Figure 1f).

| HiBiT-TDS | Normalized luminescence (%) | Reduction (%) | p value vs control |
| --- | --- | --- | --- |
| - | 100 ± 3.6 | 0 |  |
| 1 | 68 ± 1.3 | 32.0 | 0.03 (*) |
| 2 | 200.1 ± 5.5 | -100.1 | <0.001 (***) |
| 3 | 73.2 ± 5.5 | 26.8 | 0.1 (ns) |
| 4 | 84.3 ± 2.1 | 15.7 | 0.64 (ns) |
| 5 | 117.3 ± 14.1 | -17.3 | 0.53 (ns) |
| 6 | 127.8 ± 3.2 | -27.8 | 0.08 (ns) |
| 7 | 106.6 ± 9.3 | -6.6 | >0.99 (ns) |
| 8 | 174.4 ± 11.7 | -74.4 | <0.001 (***) |
| 9 | 116.3 ± 4.6 | -16.3 | 0.61 (ns) |
| 10 | 78.8 ± 2.3 | 21.2 | 0.3 (ns) |
| 11 | 86.5 ± 9.5 | 13.5 | 0.8 (ns) |
| 12 | 84.6 ± 8.5 | 15.4 | 0.67 (ns) |
| 13 | 75 ± 1.1 | 25.0 | 0.15 (ns) |
| 14 | 71.7 ± 7.6 | 28.3 | 0.08 (ns) |

Values represent normalized luminescence signal (%, mean ± SEM, n=3) relative to HiBiT/LgBiT control (100%). Reduction (%) = 100 − normalized signal. Statistical comparisons performed by ordinary one-way ANOVA with Dunnett's post-hoc test vs. control. In the "p value vs control" columns, the symbols shown in parentheses denote significance levels: *p < 0.05, **p < 0.01, ***p < 0.001; ns = not significant (p ≥ 0.05). SEM, standard error of the mean.

Table S3. Quantitative luminescence data for HiBiT-TDS candidates in HepG2 cells at 72 h (Figure 1g).

| HiBiT-TDS | Normalized luminescence (%) | Reduction (%) | p value vs control |
| --- | --- | --- | --- |
| - | 100 ± 2.2 | 0.0 |  |
| 1 | 44.5 ± 2.7 | 55.5 | <0.001 (***) |
| 2 | 100.5 ± 11.6 | -0.5 | >0.99 (ns) |
| 3 | 65.1 ± 3.8 | 34.9 | 0.006 (**) |
| 4 | 59.8 ± 5.1 | 40.2 | 0.001 (**) |
| 5 | 103.4 ± 15.5 | -3.4 | >0.99 (ns) |
| 6 | 62.7 ± 1.4 | 37.3 | 0.003 (**) |
| 7 | 78.1 ± 2.9 | 21.9 | 0.17 (ns) |
| 8 | 65.4 ± 0.3 | 34.6 | 0.007 (**) |
| 9 | 142.3 ± 10 | -42.3 | <0.001 (***) |
| 10 | 44.4 ± 1.6 | 55.6 | <0.001 (***) |
| 11 | 63.1 ± 0.6 | 36.9 | 0.003 (**) |
| 12 | 69.8 ± 3.9 | 30.2 | 0.02 (*) |
| 13 | 67.4 ± 2.8 | 32.6 | 0.01 (*) |
| 14 | 70.7 ± 7 | 29.3 | 0.03 (*) |

Values represent normalized luminescence signal (%, mean ± SEM, n=3) relative to HiBiT/LgBiT control (100%). Reduction (%) = 100 − normalized signal. Statistical comparisons performed by ordinary one-way ANOVA with Dunnett's post-hoc test vs. control. In the "p value vs control" columns, the symbols shown in parentheses denote significance levels: *p < 0.05, **p < 0.01, ***p < 0.001; ns = not significant (p ≥ 0.05). SEM, standard error of the mean.

Table S4. Quantitative luminescence data for HiBiT-TDS candidates in HepG2 cells at 120 h (Figure 1g).

| HiBiT-TDS | Normalized luminescence (%) | Reduction (%) | p value vs control |
| --- | --- | --- | --- |
| - | 100 ± 2.2 | 0.0 |  |
| 1 | 55.1 ± 3.1 | 44.9 | 0.001 (**) |
| 2 | 102.3 ± 6.2 | -2.3 | >0.99 (ns) |
| 3 | 60.2 ± 12.1 | 39.8 | 0.005 (**) |
| 4 | 68.2 ± 5.3 | 31.8 | 0.04 (*) |
| 5 | 107.2 ± 9.4 | -7.2 | >0.99 (ns) |
| 6 | 66.6 ± 4.5 | 33.4 | 0.03 (*) |
| 7 | 116.3 ± 12.6 | -16.3 | 0.61 (ns) |
| 8 | 108.4 ± 6.7 | -8.4 | >0.99 (ns) |
| 9 | 156.7 ± 4.8 | -56.7 | <0.001 (***) |
| 10 | 54.3 ± 6 | 45.7 | 0.001 (**) |
| 11 | 75.1 ± 2.9 | 24.9 | 0.16 (ns) |
| 12 | 77.8 ± 4.1 | 22.2 | 0.26 (ns) |
| 13 | 75.8 ± 6.1 | 24.2 | 0.18 (ns) |
| 14 | 76.9 ± 10.7 | 23.1 | 0.22 (ns) |

Values represent normalized luminescence signal (%, mean ± SEM, n=3) relative to HiBiT/LgBiT control (100%). Reduction (%) = 100 − normalized signal. Statistical comparisons performed by ordinary one-way ANOVA with Dunnett's post-hoc test vs. control. In the "p value vs control" columns, the symbols shown in parentheses denote significance levels: *p < 0.05, **p < 0.01, ***p < 0.001; ns = not significant (p ≥ 0.05). SEM, standard error of the mean.

Table S5. Quantitative luminescence data for HiBiT-TDP candidates in HeLa cells (Figure 2a).

| HiBiT-TDP | Normalized luminescence (%) | Reduction (%) | p value vs control |
| --- | --- | --- | --- |
| EGFP | 100 ± 6.4 | 0.0 |  |
| UBE2B | 73 ± 3.3 | 27.0 | <0.001 (***) |
| FBXL12 | 64.6 ± 1.8 | 35.4 | <0.001 (***) |
| FBXL15 | 62.5 ± 0.4 | 37.5 | <0.001 (***) |
| SPOP | 69.4 ± 0.8 | 30.6 | <0.001 (***) |
| EID1 | 63.7 ± 0.7 | 36.3 | <0.001 (***) |
| PRR20A | 48.2 ± 1.4 | 51.8 | <0.001 (***) |
| FCGR3B | 39.5 ± 1 | 60.5 | <0.001 (***) |
| LY6D | 28.3 ± 1.7 | 71.7 | <0.001 (***) |
| GABARAP | 52.2 ± 3.7 | 47.8 | <0.001 (***) |

Values represent normalized luminescence signal (%, mean ± SEM, n=3) relative to HiBiT/LgBiT control (100%). Reduction (%) = 100 − normalized signal. Statistical comparisons performed by ordinary one-way ANOVA with Dunnett's post-hoc test vs. control. In the "p value vs control" columns, the symbols shown in parentheses denote significance levels: *p < 0.05, **p < 0.01, ***p < 0.001; ns = not significant (p ≥ 0.05). SEM, standard error of the mean.

Table S6. Quantitative luminescence data for HiBiT-TDP candidates in HepG2 cells (Figure 2a).

| HiBiT-TDP | Normalized luminescence (%) | Reduction (%) | p value vs control |
| --- | --- | --- | --- |
| EGFP | 100 ± 10.9 | 0.0 |  |
| UBE2B | 123.7 ± 1.9 | -23.7 | 0.17 (ns) |
| FBXL12 | 33.8 ± 2.8 | 66.2 | <0.001 (***) |
| FBXL15 | 55.8 ± 7.2 | 44.2 | 0.003 (**) |
| SPOP | 32.9 ± 4.1 | 67.1 | <0.001 (***) |
| EID1 | 64.5 ± 11.7 | 35.5 | 0.02 (*) |
| PRR20A | 58.7 ± 5.3 | 41.3 | 0.005 (**) |
| FCGR3B | 13 ± 1 | 87.0 | <0.001 (***) |
| LY6D | 8.4 ± 0.7 | 91.6 | <0.001 (***) |
| GABARAP | 122.8 ± 12.8 | -22.8 | 0.21 (ns) |

Values represent normalized luminescence signal (%, mean ± SEM, n=3) relative to HiBiT/LgBiT control (100%). Reduction (%) = 100 − normalized signal. Statistical comparisons performed by ordinary one-way ANOVA with Dunnett's post-hoc test vs. control. In the "p value vs control" columns, the symbols shown in parentheses denote significance levels: *p < 0.05, **p < 0.01, ***p < 0.001; ns = not significant (p ≥ 0.05). SEM, standard error of the mean.

Table S7. Quantitative Western blotting analysis data for HiBiT-TDP candidates in HepG2 cells (Figure 2b, 2c).

| HiBiT-TDP | LgBiT protein reduction (%) | p value vs control |
| --- | --- | --- |
| UBE2B | 2.5 ± 13 | >0.99 (ns) |
| SPOP | 82.3 ± 6.8 | <0.001 (***) |
| EID1 | 69.6 ± 2.5 | 0.002 (**) |
| PRR20A | 31 ± 9.5 | 0.25 (ns) |
| FCGR3B | 65.1 ± 1.5 | 0.003 (**) |
| LY6D | 59.8 ± 24.2 | 0.007 (**) |
| GABARAP | 60.6 ± 5.8 | 0.006 (**) |

Values represent LgBiT protein reduction (%, mean ± SEM, n=3) relative to the mock, calculated as 100 × (1 - relative protein fold), where the relative protein fold is the loading-control-normalized band intensity expressed as fold change versus the mock. Statistical comparisons were performed by ordinary one-way ANOVA with Dunnett's post-hoc test versus control, using GraphPad Prism 10. In the "p value vs control" columns, the symbols shown in parentheses denote significance levels: *p < 0.05, **p < 0.01, ***p < 0.001; ns = not significant (p ≥ 0.05). SEM, standard error of the mean.

Table S8. Quantitative Western blotting analysis data for HiBiT-TDP candidates in HeLa cells (Figure S1a, S1b).

| HiBiT-TDP | LgBiT protein reduction (%) | p value vs control |
| --- | --- | --- |
| SPOP | 61.2 ± 5.5 | <0.001 (***) |
| EID1 | 52.1 ± 6 | 0.004 (**) |
| FCGR3B | 56.5 ± 8.8 | 0.001 (**) |
| LY6D | 56.2 ± 6.2 | 0.002 (**) |
| GABARAP | 52.2 ± 14.8 | 0.004 (**) |

Values represent LgBiT protein reduction (%, mean ± SEM, n=3) relative to the mock, calculated as 100 × (1 - relative protein fold), where the relative protein fold is the loading-control-normalized band intensity expressed as fold change versus the mock. Statistical comparisons were performed by ordinary one-way ANOVA with Dunnett's post-hoc test versus control, using GraphPad Prism 10. In the "p value vs control" columns, the symbols shown in parentheses denote significance levels: *p < 0.05, **p < 0.01, ***p < 0.001; ns = not significant (p ≥ 0.05). SEM, standard error of the mean.

Table S9. Quantitative fluorescence data for vhhGFP-TDP candidates in HeLa cells (Figure 2e).

| vhhGFP-TDP | Normalized fluorescence (%) | Reduction (%) | p value vs control |
| --- | --- | --- | --- |
| Mock | 100 ± 1.9 | 0 |  |
| UBE2B | 46.9 ± 2.3 | 53.1 | <0.001 (***) |
| FBXL12 | 72.3 ± 6.8 | 27.7 | 0.04 (*) |
| FBXL15 | 72 ± 5.7 | 28 | 0.04 (*) |
| SPOP | 89.2 ± 7.9 | 10.8 | 0.79 (ns) |
| EID1 | 28.7 ± 2.3 | 71.3 | <0.001 (***) |
| PRR20A | 146.4 ± 15.5 | -46.4 | <0.001 (***) |
| FCGR3B | 36.7 ± 0.8 | 63.3 | <0.001 (***) |
| LY6D | 16.6 ± 0.9 | 83.4 | <0.001 (***) |
| GABARAP | 35.2 ± 3.3 | 64.8 | <0.001 (***) |

Values represent normalized luminescence signal (%, mean ± SEM, n=3) relative to HiBiT/LgBiT control (100%). Reduction (%) = 100 − normalized signal. Statistical comparisons performed by ordinary one-way ANOVA with Dunnett's post-hoc test vs. control. In the "p value vs control" columns, the symbols shown in parentheses denote significance levels: *p < 0.05, **p < 0.01, ***p < 0.001; ns = not significant (p ≥ 0.05). SEM, standard error of the mean.

Table S10. Quantitative fluorescence data for vhhGFP-TDP candidates in HepG2 cells (Figure 2e).

| vhhGFP-TDP | Normalized fluorescence (%) | Reduction (%) | p value vs control |
| --- | --- | --- | --- |
| Mock | 100 ± 4.4 | 0.0 |  |
| UBE2B | 55.8 ± 4.4 | 44.2 | 0.07 (ns) |
| FBXL12 | 77.1 ± 13.5 | 22.9 | 0.62 (ns) |
| FBXL15 | 75.6 ± 27.1 | 24.4 | 0.56 (ns) |
| SPOP | 49.9 ± 5.2 | 50.1 | 0.03 (*) |
| EID1 | 37.1 ± 2.9 | 62.9 | 0.005 (**) |
| PRR20A | 175.5 ± 13.2 | -75.5 | <0.001 (***) |
| FCGR3B | 24.6 ± 2.6 | 75.4 | <0.001 (***) |
| LY6D | 44.2 ± 1 | 55.8 | 0.01 (*) |
| GABARAP | 72.2 ± 8.7 | 27.8 | 0.42 (ns) |

Values represent normalized luminescence signal (%, mean ± SEM, n=3) relative to HiBiT/LgBiT control (100%). Reduction (%) = 100 − normalized signal. Statistical comparisons performed by ordinary one-way ANOVA with Dunnett's post-hoc test vs. control. In the "p value vs control" columns, the symbols shown in parentheses denote significance levels: *p < 0.05, **p < 0.01, ***p < 0.001; ns = not significant (p ≥ 0.05). SEM, standard error of the mean.

Table S11. Quantitative Western blotting analysis data for vhhGFP-TDP candidates in HepG2 cells (Figure 2f, 2g).

| vhhGFP-TDP | EGFP protein reduction (%) | p value vs control |
| --- | --- | --- |
| UBE2B | 47.2 ± 4.1 | 0.11 (ns) |
| SPOP | 20.3 ± 8.3 | 0.81 (ns) |
| EID1 | 57.5 ± 22.6 | 0.04 (*) |
| PRR20A | -47.3 ± 27.6 | 0.11 (ns) |
| FCGR3B | 70.4 ± 1.7 | 0.01 (*) |
| LY6D | 66.3 ± 7.1 | 0.02 (*) |
| GABARAP | 60.1 ± 3.2 | 0.03 (*) |

Values represent EGFP protein reduction (%, mean ± SEM, n=3) relative to the mock, calculated as 100 × (1 - relative protein fold), where the relative protein fold is the loading-control-normalized band intensity expressed as fold change versus the mock. Statistical comparisons were performed by ordinary one-way ANOVA with Dunnett's post-hoc test versus control, using GraphPad Prism 10. In the "p value vs control" columns, the symbols shown in parentheses denote significance levels: *p < 0.05, **p < 0.01, ***p < 0.001; ns = not significant (p ≥ 0.05). SEM, standard error of the mean.

Table S12. Quantitative Western blotting analysis data for vhhGFP-TDP candidates in HeLa cells (Figure S1c, S1d).

| vhhGFP-TDP | EGFP protein reduction (%) | p value vs control |
| --- | --- | --- |
| SPOP | 42.9 ± 14 | 0.03 (*) |
| EID1 | 76.1 ± 4.7 | <0.001 (***) |
| PRR20A | -88.9 ± 17.8 | <0.001 (***) |
| FCGR3B | 57.3 ± 6.3 | 0.004 (**) |
| LY6D | 72.7 ± 3.9 | <0.001 (***) |
| GABARAP | 64.2 ± 8 | 0.002 (**) |

Values represent EGFP protein reduction (%, mean ± SEM, n=3) relative to the mock, calculated as 100 × (1 - relative protein fold), where the relative protein fold is the loading-control-normalized band intensity expressed as fold change versus the mock. Statistical comparisons were performed by ordinary one-way ANOVA with Dunnett's post-hoc test versus control, using GraphPad Prism 10. In the "p value vs control" columns, the symbols shown in parentheses denote significance levels: *p < 0.05, **p < 0.01, ***p < 0.001; ns = not significant (p ≥ 0.05). SEM, standard error of the mean.

Table S13. Quantitative fluorescence data for vhhGFP-TDS candidates in HeLa cells (Figure 2h).

| vhhGFP-TDS | Normalized fluorescence (%) | Reduction (%) | p value vs control |
| --- | --- | --- | --- |
| - | 100 ± 4.1 | 0.0 |  |
| 1 | 54.6 ± 2.3 | 45.4 | <0.001 (***) |
| 2 | 101.6 ± 10.4 | -1.6 | >0.99 (ns) |
| 3 | 98.7 ± 8.6 | 1.3 | >0.99 (ns) |
| 5 | 95.8 ± 3.8 | 4.2 | >0.99 (ns) |
| 9 | 92.8 ± 5.6 | 7.2 | 0.96 (ns) |
| 10 | 106.6 ± 7.8 | -6.6 | 0.97 (ns) |
| 13 | 90.6 ± 4.8 | 9.4 | 0.87 (ns) |
| 14 | 91.7 ± 6.8 | 8.3 | 0.92 (ns) |

Table S14. Quantitative fluorescence data for vhhGFP-TDS candidates in HepG2 cells (Figure 2h).

| vhhGFP-TDS | Normalized fluorescence (%) | Reduction (%) | p value vs control |
| --- | --- | --- | --- |
| - | 100 ± 5.5 | 0.0 |  |
| 1 | 64.3 ± 3.5 | 35.7 | 0.01 (*) |
| 2 | 81.4 ± 4.9 | 18.6 | 0.32 (ns) |
| 3 | 72.9 ± 0.9 | 27.1 | 0.07 (ns) |
| 5 | 60.5 ± 6.8 | 39.5 | 0.005 (**) |
| 9 | 92.8 ± 7.9 | 7.2 | 0.97 (ns) |
| 10 | 82.8 ± 12.7 | 17.2 | 0.4 (ns) |
| 13 | 70.9 ± 5.8 | 29.1 | 0.05 (*) |
| 14 | 85.8 ± 7.8 | 14.2 | 0.6 (ns) |

Values represent normalized fluorescence signal (%, mean ± SEM, n=3) relative to vhhGFP/ EGFP control (100%). Reduction (%) = 100 − normalized signal. Statistical comparisons performed by ordinary one-way ANOVA with Dunnett's post-hoc test vs. control. In the "p value vs control" columns, the symbols shown in parentheses denote significance levels: *p < 0.05, **p < 0.01, ***p < 0.001; ns = not significant (p ≥ 0.05). SEM, standard error of the mean.

Table S15. Quantitative luminescence data for HiBiT-TDP candidates in HeLa-LgBiT-EGFP-NLS stable cells (Figure 3b).

| HiBiT-TDP | Normalized luminescence (%) | Reduction (%) | p value vs control |
| --- | --- | --- | --- |
| Mock | 100 ± 10.3 | 0 |  |
| UBE2B | 57.3 ± 3.7 | 42.7 | <0.001 (***) |
| FBXL12 | 49.1 ± 2.3 | 50.9 | <0.001 (***) |
| FBXL15 | 53.1 ± 3.9 | 46.9 | <0.001 (***) |
| SPOP | 15.4 ± 1.1 | 84.6 | <0.001 (***) |
| EID1 | 56.6 ± 0.9 | 43.4 | <0.001 (***) |
| PRR20A | 41.1 ± 2.4 | 58.9 | <0.001 (***) |
| FCGR3B | 11.5 ± 0.6 | 88.5 | <0.001 (***) |
| LY6D | 4.9 ± 0.3 | 95.1 | <0.001 (***) |
| GABARAP | 52 ± 2.6 | 48.0 | <0.001 (***) |

Table S16. Quantitative fluorescence data for HiBiT-TDP candidates in HeLa-LgBiT-EGFP-NLS stable cells. (Figure 3b).

| HiBiT-TDP | Normalized fluorescence (%) | Reduction (%) | p value vs control |
| --- | --- | --- | --- |
| Mock | 100 ± 7.2 | 0 |  |
| UBE2B | 95.4 ± 13.1 | 4.6 | >0.99 (ns) |
| FBXL12 | 63.8 ± 9.5 | 36.2 | 0.02 (*) |
| FBXL15 | 53.6 ± 8.4 | 46.4 | 0.003 (**) |
| SPOP | 32.2 ± 10.7 | 67.8 | <0.001 (***) |
| EID1 | 119.7 ± 4.5 | -19.7 | 0.38 (ns) |
| PRR20A | 94 ± 5.7 | 6.0 | >0.99 (ns) |
| FCGR3B | 54.1 ± 1.7 | 45.9 | 0.003 (**) |
| LY6D | 64.4 ± 3.2 | 35.6 | 0.02 (*) |
| GABARAP | 77 ± 3.7 | 23.0 | 0.23 (ns) |

Values represent normalized luminescence or fluorescence signal (%, mean ± SEM, n=3) relative to HiBiT/LgBiT-EGFP-NLS control (100%). Reduction (%) = 100 − normalized signal. Statistical comparisons performed by ordinary one-way ANOVA with Dunnett's post-hoc test vs. control. In the "p value vs control" columns, the symbols shown in parentheses denote significance levels: *p < 0.05, **p < 0.01, ***p < 0.001; ns = not significant (p ≥ 0.05). SEM, standard error of the mean.

Table S17. Quantitative fluorescence data for vhhGFP-TDS candidates in HeLa-LgBiT-EGFP-NLS stable cells (Figure 3e).

| VHH-TDS | Normalized fluorescnece (%) | Reduction (%) | p value vs control |
| --- | --- | --- | --- |
| - | 100 ± 2.5 | 0.0 |  |
| 1 | 93.6 ± 3 | 6.4 | 0.93 (ns) |
| 2 | 111.4 ± 1.5 | -11.4 | 0.37 (ns) |
| 3 | 73.4 ± 3.2 | 26.6 | <0.001 (***) |
| 4 | 93.2 ± 0.8 | 6.8 | 0.89 (ns) |
| 5 | 97.1 ± 2.8 | 2.9 | >0.99 (ns) |
| 6 | 80.9 ± 4.4 | 19.1 | 0.02 (*) |
| 7 | 92.8 ± 0.9 | 7.2 | 0.86 (ns) |
| 8 | 105.3 ± 0.9 | -5.3 | 0.98 (ns) |
| 9 | 82.9 ± 6.8 | 17.1 | 0.06 (ns) |
| 10 | 150.7 ± 7.2 | -50.7 | <0.001 (***) |
| 11 | 119.4 ± 5.8 | -19.4 | 0.02 (*) |
| 12 | 107.7 ± 3.2 | -7.7 | 0.8 (ns) |
| 13 | 103.4 ± 4.2 | -3.4 | >0.99 (ns) |
| 14 | 106.7 ± 5.9 | -6.7 | 0.9 (ns) |

Values represent normalized fluorescence signal (%, mean ± SEM, n=3) relative to vhhGFP/ EGFP control (100%). Reduction (%) = 100 − normalized signal. Statistical comparisons performed by ordinary one-way ANOVA with Dunnett's post-hoc test vs. control. In the "p value vs control" columns, the symbols shown in parentheses denote significance levels: *p < 0.05, **p < 0.01, ***p < 0.001; ns = not significant (p ≥ 0.05). SEM, standard error of the mean.

Table S18. Quantitative fluorescence data for vhhGFP-TDP candidates in HeLa-LgBiT-EGFP-NLS stable cells (Figure 3e).

| vhhGFP-TDP | Normalized fluorescence (%) | Reduction (%) | p value vs control |
| --- | --- | --- | --- |
| - | 100 ± 2 | 0.0 |  |
| UBE2B | 73.6 ± 1.6 | 26.4 | <0.001 (***) |
| FBXL12 | 42 ± 0.5 | 58.0 | <0.001 (***) |
| FBXL15 | 47.2 ± 1.7 | 52.8 | <0.001 (***) |
| SPOP | 24.8 ± 0.7 | 75.2 | <0.001 (***) |
| EID1 | 34.2 ± 1.2 | 65.8 | <0.001 (***) |
| PRR20A | 41.8 ± 0.2 | 58.2 | <0.001 (***) |
| FCGR3B | 34.7 ± 0.8 | 65.3 | <0.001 (***) |
| LY6D | 31.8 ± 0.9 | 68.2 | <0.001 (***) |
| GABARAP | 49.6 ± 2.9 | 50.4 | <0.001 (***) |

Values represent normalized fluorescence signal (%, mean ± SD, n=3) relative to vhhGFP/ EGFP control (100%). Reduction (%) = 100 − normalized signal. Statistical comparisons performed by ordinary one-way ANOVA with Dunnett's post-hoc test vs. control. In the "p value vs control" columns, the symbols shown in parentheses denote significance levels: *p < 0.05, **p < 0.01, ***p < 0.001; ns = not significant (p ≥ 0.05). SEM, standard error of the mean.

Table S19. Comparison of protein degradation assay platforms in this study

**Supplemental figures**

**
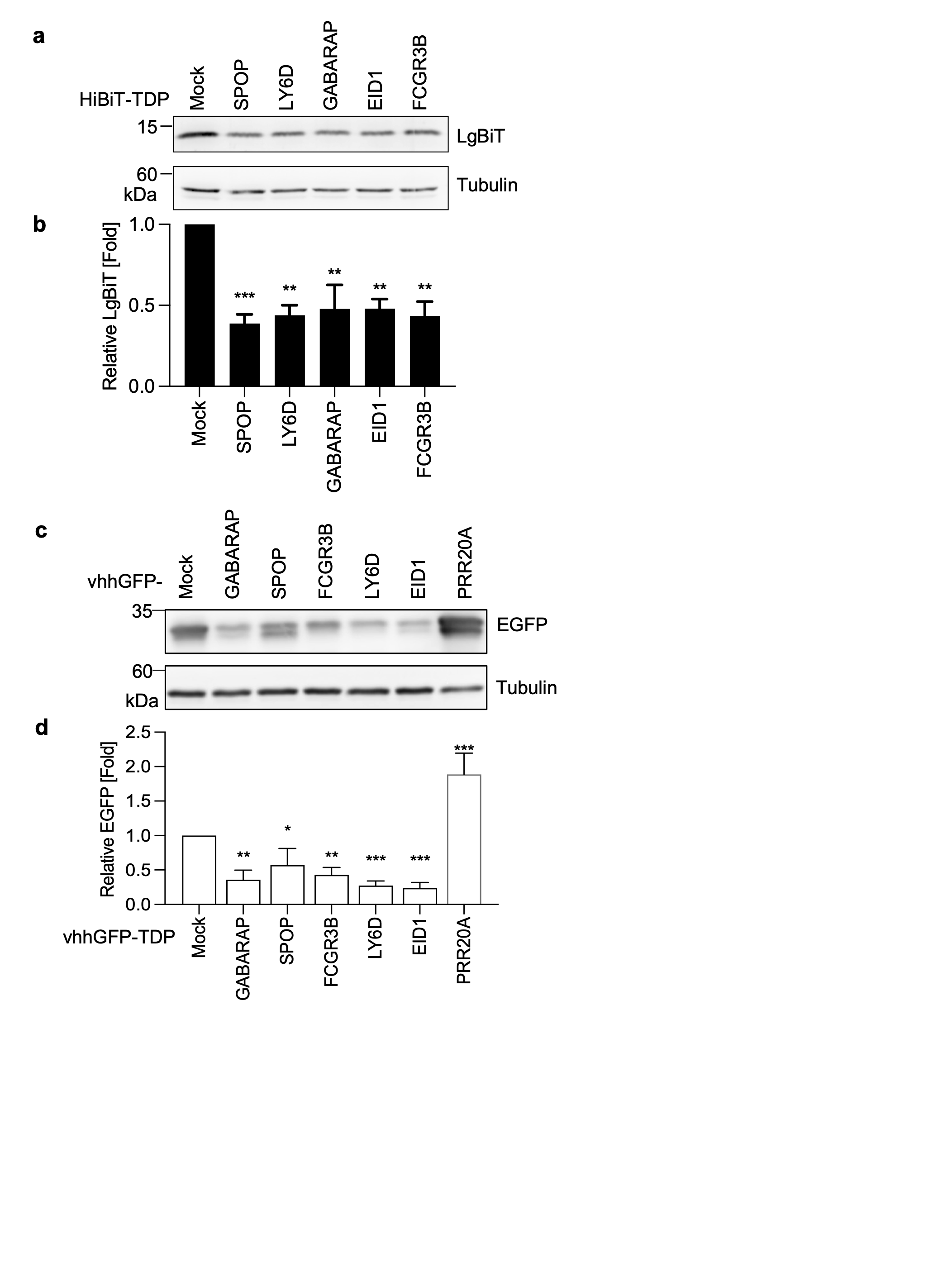
**

*Figure S1. Western blot validation of HiBiT-TDP and vhhGFP-TDP mediated degradation in HeLa cells.*

(a) Representative Western blot images of LgBiT protein levels in HeLa cells co-transfected with the HiBiT-TDP constructs. Tubulin served as the loading control. (b) Corresponding densitometric quantification of LgBiT band intensity from (a), normalized to tubulin and expressed as fold change relative to the mock. Data are shown as mean ± SEM from three independent experiments (n = 3). Statistical significance was determined by one-way ANOVA followed by Dunnett's multiple comparisons test against the mock. (c) Representative Western blot images of EGFP protein levels in HeLa cells co-transfected with the vhhGFP-TDP constructs. Tubulin served as the loading control. (d) Corresponding densitometric quantification of EGFP band intensity from (c), normalized to tubulin and expressed as fold change relative to the mock. Data are shown as mean ± SEM from three independent experiments (n = 3). Statistical significance was determined by one-way ANOVA followed by Dunnett's multiple comparisons test against the mock (GraphPad Prism10). Statistical significance is indicated as follows: ∗P < 0.05; ∗∗P < 0.01; ∗∗∗P < 0.001.


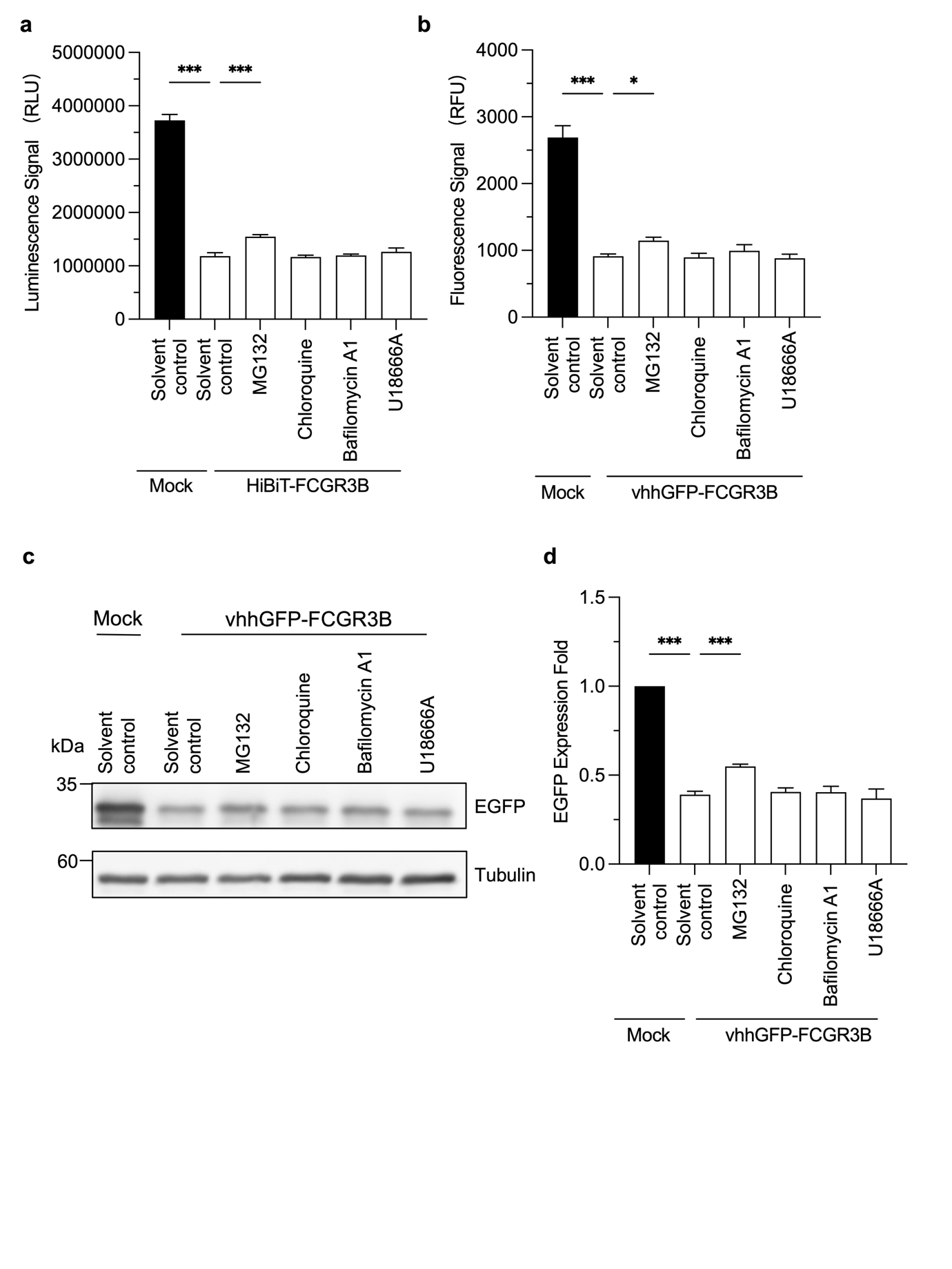


*Figure S2. Inhibitor-based pathway validation of FCGR3B-mediated degradation*

(a) NanoBiT-based degradation assay in HeLa cells co-expressing LgBiT and HiBiT-FCGR3B. Cells were treated with proteasome inhibitor (MG132) or lysosomal inhibitors (bafilomycin A1, chloroquine, and U18666A) to evaluate degradation pathway involvement. Luminescence signals were measured as a readout of LgBiT abundance. Data are presented as mean ± SEM (n = 3). (b) Anti-GFP nanobody (vhhGFP)-based degradation assay in HeLa cells co-expressing enhanced green fluorescent protein (EGFP) and vhhGFP-FCGR3B. Fluorescence intensity was quantified following inhibitor treatment. Data are presented as mean ± SEM (n = 3). (c) Representatives Western blot images of EGFP levels in HeLa cells expressing vhhGFP-FCGR3B under the indicated inhibitor treatments. (d) Densitometric quantification of Western blot signals shown in (c). Band intensities were normalized to the corresponding loading control and expressed as fold change relative to mock. Data are presented as mean ± SEM (n = 3). Data are representative of at least two independent experiments. Statistical analyses were performed using ordinary one-way ANOVA followed by Šídák's multiple comparisons test (GraphPad Prism 10). Statistical significance is indicated as follows: ∗P < 0.05; ∗∗P < 0.01; ∗∗∗P < 0.001.


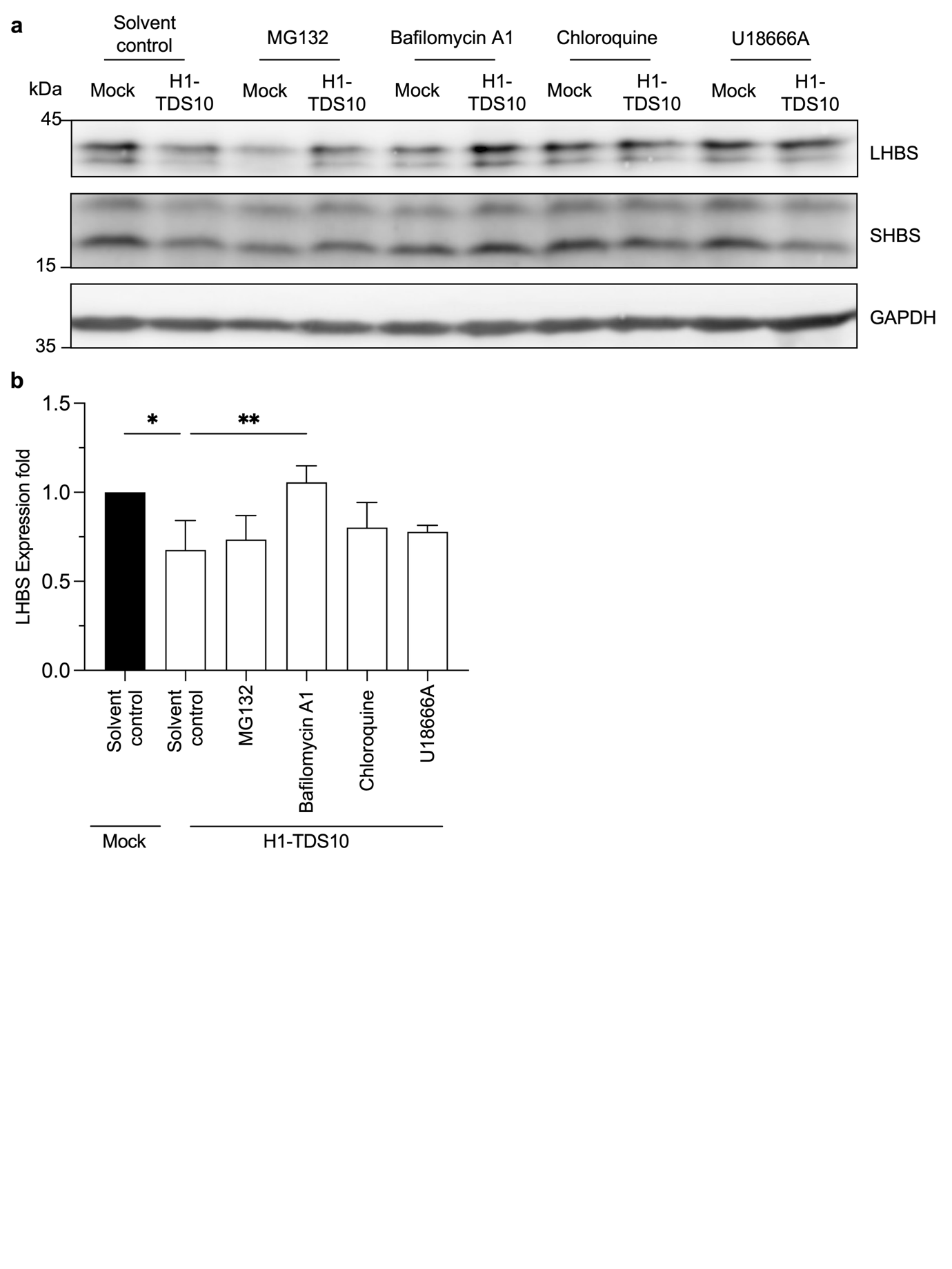


*Figure S3. Inhibitor validation of H1-TDS#10-mediated degradation in Hepatitis B virus (HBV) model*

(a) Representatives Western blot images of large hepatitis B surface antigen (LHBS) protein levels in HepG2 cells co-transfected with the pHBV3.6 replicon and H1-TDS#10 construct, followed by treatment with the indicated inhibitors for 6 h prior to analysis. (b) Densitometric quantification of Western blot signals shown in (a). Band intensities were normalized to the corresponding loading control and expressed as fold change relative to mock. Treatment with the lysosomal inhibitor bafilomycin A1 significantly restored LHBS protein levels, supporting a lysosome-dependent degradation mechanism for H1-TDS#10. Data are presented as mean ± SEM (n = 3). Data are representative of at least two independent experiments. Statistical analyses were performed using ordinary one-way ANOVA followed by Šídák's multiple comparisons test (GraphPad Prism 10). Statistical significance is indicated as follows: ∗∗P < 0.01; ns, not significant.


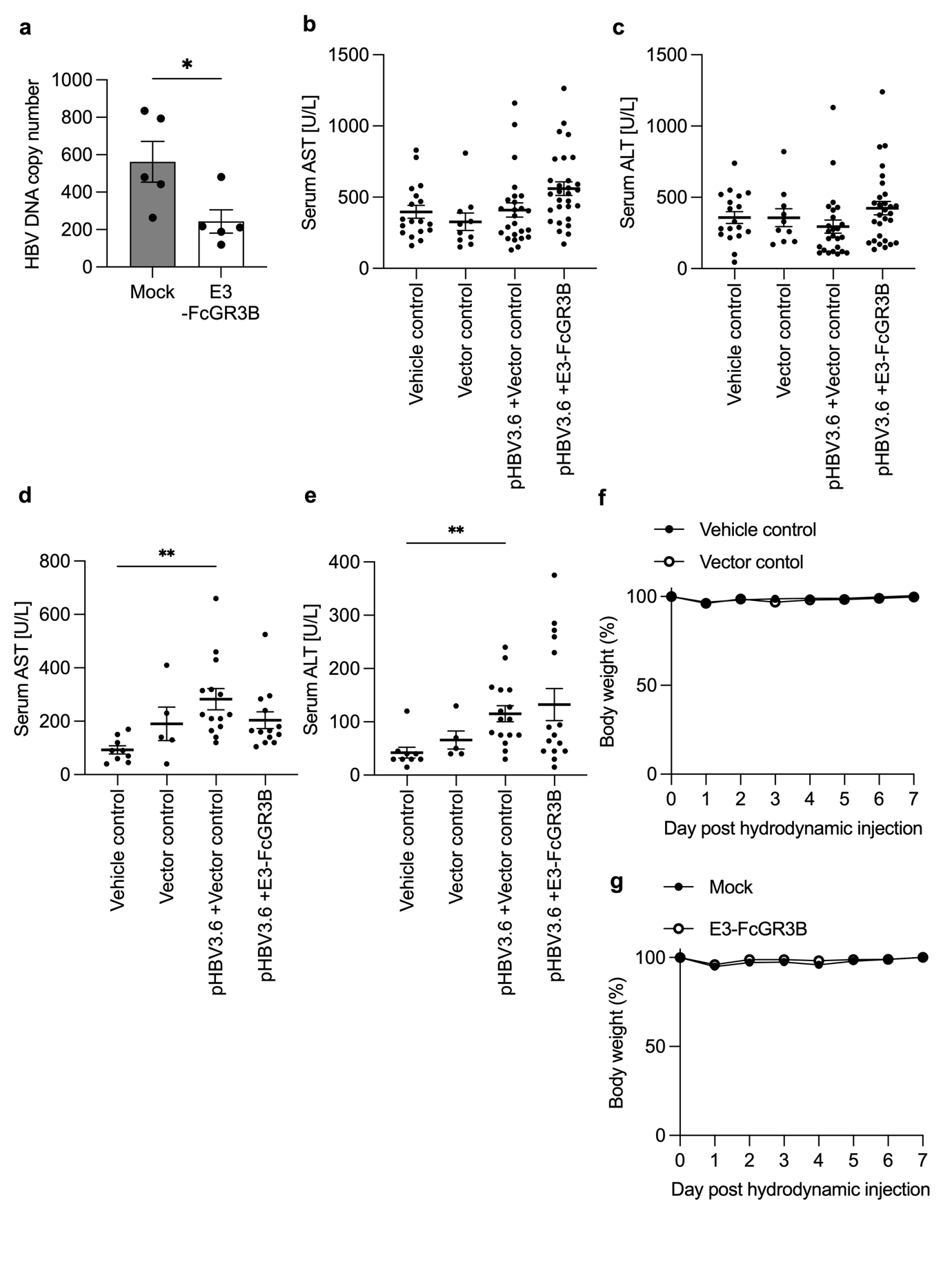


*Figure S4. In vivo evaluation of antiviral efficacy and liver toxicity markers.*

(a) Serum HBV DNA copy number quantified by quantitative PCR (qPCR) at Day 7., showing a significant reduction in viral DNA levels in the E3-FCGR3B-treated group compared with vector control. Data are presented as mean ± SEM (n = 5). (b, c) Serum aspartate aminotransferase (AST) (b) and alanine aminotransferase (ALT) (c) levels measured at Day 2. No significant differences were observed among experimental groups, indicating minimal acute hepatotoxicity. (d, e) Serum AST (d) and ALT (e) levels measured at Day 7 Although the vehicle control group showed lower enzyme levels compared with the mock group, no significant differences were observed between mock and E3-FCGR3B-treated groups. (f) Body weight of mice in the vehicle control group (Day 2, n = 23; Day 7, n = 9), vector control group (Day 2 and Day 7, n = 10 each), (g) mock group (pHBV3.6 + vector; Day 2., n = 41; Day 7., n = 21), and E3-FCGR3B-treated group (Day 2., n = 35; Day 7., n = 20) was recorded daily from Day 0 (hydrodynamic injection) through the experimental endpoint. Data are expressed as percentage of initial body weight on Day 0 and presented as mean ± SEM. No animal in any group exhibited body weight loss exceeding 15% of initial body weight at any time point during the study, in compliance with institutional humane endpoint criteria. Data are representative of at least two independent experiments. Statistical analyses were performed as follows: data in (a) were analyzed using an unpaired two-tailed Student's t-test. Data in (b) and (c) were analyzed using ordinary one-way ANOVA followed by Tukey's multiple comparisons test. Data in (d) and (e) were analyzed using Brown-Forsythe and Welch's ANOVA followed by Dunnett's T3 multiple comparisons test (GraphPad Prism 10). Statistical significance is indicated as follows: ∗P < 0.05; ∗∗P < 0.01; ns, not significant.


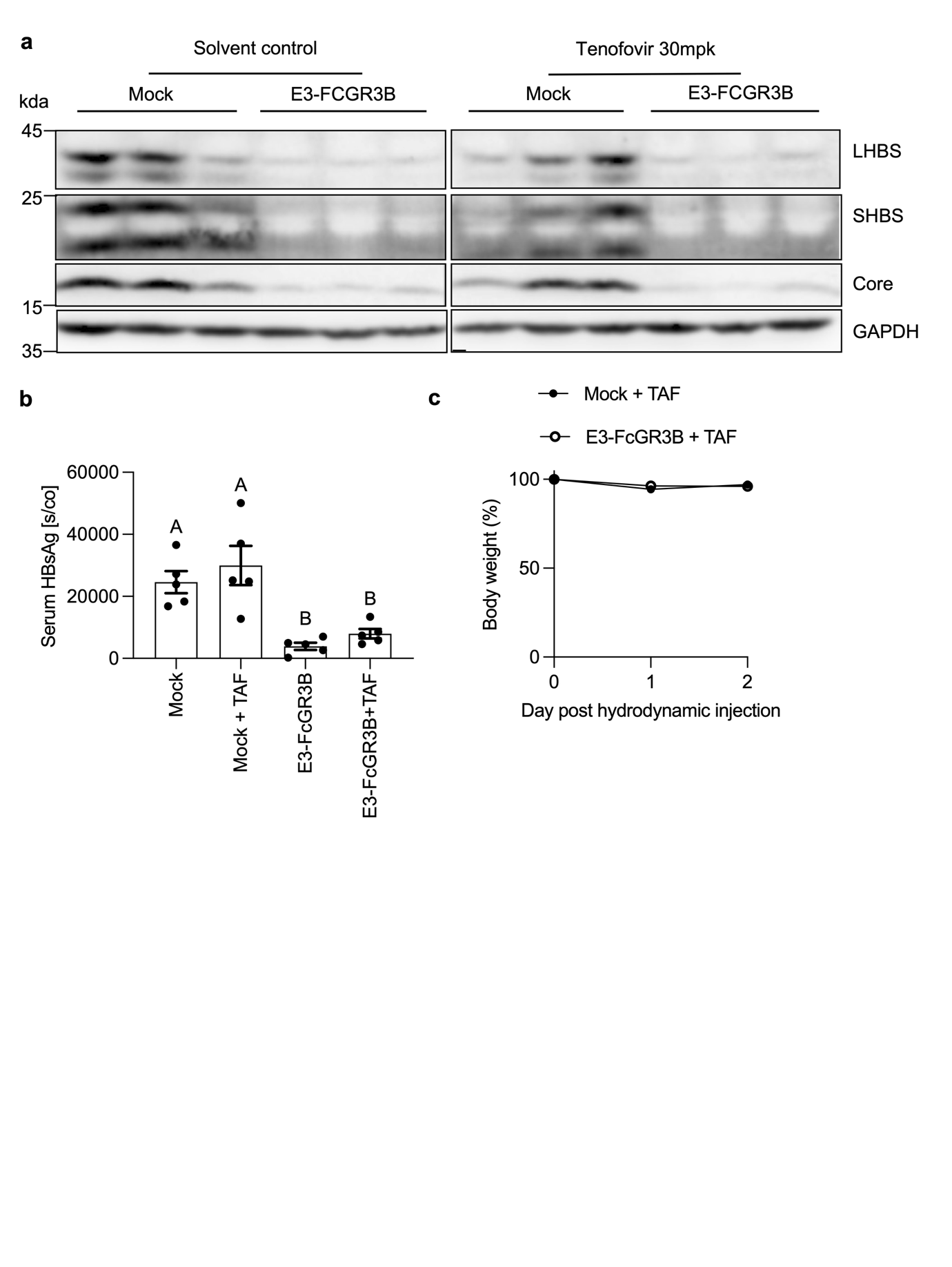


*Figure S5. Evaluation of tenofovir alafenamide (TAF) co-treatment in the HBV HDI mouse model.*

(a) Representatives Western blot images of intrahepatic HBV proteins (LHBS, SHBS, and core) at day 2, following a single oral administration of TAF at day 0. No detectable reduction in viral protein levels was observed under these conditions (n = 5). (b) Serum HBsAg levels at day 2. Compact letter display indicates no statistically significant difference between mock and mock + TAF groups, whereas E3-FCGR3B-treated groups showed significant reduction compared with mock controls. (c) Body weight of mice in the mock + TAF group and E3-FCGR3B group (both 2 days., n = 5 per group) was recorded daily from day 0 through the experimental endpoint. Data are presented as mean ± SEM and expressed as percentage of initial body weight on day 0. No animal exhibited body weight loss exceeding 15% of initial body weight at any time point during the study, in compliance with institutional humane endpoint criteria. These results suggest that, under the experimental conditions tested, TAF does not significantly impact HBV antigen levels, in contrast to the direct protein degradation mediated by Nab-TAC. Statistical analyses were performed using ordinary one-way ANOVA followed by Turkey’s post hoc multiple comparison tests (GraphPad Prism 10).

**Appendix-1**

**Abbreviation List**

| **Abbreviation** | **Full Term** |
| --- | --- |
| ALT | Alanine aminotransferase |
| AST | Aspartate aminotransferase |
| cccDNA | Covalently closed circular DNA |
| EGFP | Enhanced green fluorescent protein |
| FCGR3B | Fc gamma receptor IIIb |
| GAPDH | Glyceraldehyde-3-phosphate dehydrogenase |
| HBcAg | Hepatitis B core antigen |
| HBV | Hepatitis B virus |
| HBeAg | Hepatitis B e antigen |
| HBsAg | Hepatitis B surface antigen |
| HDI | Hydrodynamic injection |
| IHC | Immunohistochemistry |
| LHBS | Large hepatitis B surface antigen |
| LNP | Lipid nanoparticle |
| Nab-TAC | Nanobody-based targeted protein degradation chimera |
| NanoBiT | NanoLuc Binary Technology |
| NLS | Nuclear localization signal |
| qPCR | Quantitative polymerase chain reaction |
| scFv | Single-chain variable fragment |
| SEM | Standard error of the mean |
| SHBS | Small hepatitis B surface antigen |
| SPOP | Speckle-type POZ protein |
| TAF | Tenofovir alafenamide |
| TDP | Target degradation protein |
| TDS | Target degradation signal |
| TRD | Target recognition domain |
| VHH | Variable domain of heavy-chain-only antibody |
| vhhGFP | Anti-EGFP variable domain of heavy-chain-only antibody |
